# Supplementary material for: Ebola Entry Inhibitors Discovered from Maesa perlarius
Source: Int J Mol Sci. 2022 Feb 27;23(5):2620. doi: 10.3390/ijms23052620 (PMC8910447; doi:10.3390/ijms23052620)
Supplement: Supplementary file 1 [file ijms-23-02620-s001.zip › ijms-1559689-supplementary.pdf]

Ebola Entry Inhibitors Discovered from *Maesa perlarius*

Nga Yi Tsang [1,†], Wan-Fei Li [1,†], Elizabeth Varhegyi[2,†], Lijun Rong [2,*] and Hong-Jie Zhang [1,*]

[1] Teaching and Research Division, School of Chinese Medicine, Hong Kong Baptist University, Kowloon, Hong Kong SAR, China; 12012173@life.hkbu.edu.hk (N.Y.T.); wanfeier@live.cn (W.-F.L.)
[2] Department of Microbiology and Immunology, College of Medicine, University of Illinois Chicago, 909 South Wolcott Ave, Chicago, Illinois 60612, United States; estark2434@gmail.com
* Correspondence: zhanghj@hkbu.edu.hk (H.-J.Z.); Tel.: +852 34112956; lijun@uic.edu (L.R.); Tel.: +1 312-3550203
† These authors contribute equally to this work.

Supplementary Materials

Table of Contents

Table S1. Preparative HPLC condition to separate compounds from *M. perlarius* fractions 4
Figure S1. The chromatograms of preparative HPLC for fractions F64-B19, F64-B20 and F64-B1617 recorded at UV 210 nm 4
Figure S2. UPLC chromatograms of compounds **1-5** isolated from fractions B19 and B20 recorded at UV 210 nm 5
Figure S3. MS spectra of compounds **1-5** 6
Table S2. Comparison of the MS data of **1-5** and their reported literature data 7
Figure S4. MS^2 spectra of **1-5** 8
Figure S5. ^{1}H NMR spectrum of **1** in CD_3OD (400 MHz) 9
Figure S6. ^{1}H NMR spectrum of **2** in CD_3OD (400 MHz) 9
Figure S7. ^{1}H NMR spectrum of **3** in CD_3OD (400 MHz) 10
Table S3. Comparison of ^{1}H NMR spectral data of the literature procyanidin B2 and compound **1** 11
Table S4. Comparison of ^{1}H NMR spectral data of the literature procyanidin C1 and compound **2** 12
Table S5. Comparison of ^{1}H NMR spectral data of the literature procyanidin tetramer epicatechin-(4β→8)-epicatechin-(4β→8)-epicatechin-(4 β→8)-epicatechin and compound **3** 13
Figure S8. IR spectrum of **1** 14
Figure S9. IR spectrum of **2** 14
Figure S10. IR spectrum of **3** 15
Figure S11. IR spectrum of **4** 15
Figure S12. IR spectrum of **5** 16
Figure S13. UV spectrum of **1** 17
Figure S14. UV spectrum of **2** 17
Figure S15. UV spectrum of **3** 17
Figure S16. UV spectrum of **4** 18
Figure S17. UV spectrum of **5** 18
Figure S18. *In vitro* dose-response curves of plant extract fractions, flavan-3-ols and their derivatives on pseudovirion HIV-Luc/EBOV-GP. samples were evaluated in A549 cells against pseudovirion HIV-Luc/EBOV-GP 19
Figure S19.Chemical structures of flavanones showing no inhibitory effects against EBOV pseudovirus at concentrations of 50 µm 19
Figure S20. ^{1}H NMR spectrum of **12** in $CDCl_3$ (400 MHz) 20
Figure S21. ^{1}H NMR spectrum of **13a** in $CDCl_3$ (400 MHz) 21
Figure S22. ^{1}H NMR spectrum of **13b** in CD_3OD (400 MHz) 22
Figure S23. ^{1}H NMR spectrum of **14a** in $CDCl_3$ (400 MHz) 23
Figure S24. ^{13}C NMR spectrum of **14a** in $CDCl_3$ (100 MHz) 24
Figure S25. ^{1}H NMR spectrum of **14b** in acetone-d_6 (400 MHz) 25
Figure S26. ^{13}C NMR spectrum of **14b** in acetone-d_6 (100 MHz) 26
Figure S27. ^{1}H NMR spectrum of **15a** in $CDCl_3$ (400 MHz) 27
Figure S28. ^{13}C NMR spectrum of **15a** in $CDCl_3$ (100 MHz) 28
Figure S29. ^{1}H NMR spectrum of **15b** in acetone-d_6 (400 MHz) 29

Figure S30. ^{13}C NMR spectrum of **15b** in acetone-d_6 (100 MHz) 30
Figure S31. ^{1}H NMR spectrum of **16a** in $CDCl_3$ (400 MHz) 31
Figure S32. ^{13}C NMR spectrum of **16a** in $CDCl_3$ (100 MHz) 32
Figure S33. ^{1}H NMR spectrum of **16b** in acetone-d_6 (400 MHz) 33
Figure S34. ^{13}C NMR spectrum of **16b** in acetone-d_6 (100 MHz) 34
Figure S35. ^{1}H NMR spectrum of **17a** in $CDCl_3$ (400 MHz) 35
Figure S36. ^{1}H NMR spectrum of **17b** in $CDCl_3$ (400 MHz) 36
Figure S37. Binding modes of compounds docked to ebola virus glycoprotein crystal structure 5JQ7 38
Figure S38. The correlation between the docking score and the IC_{50} values for B-type procyanidins, flavan-3-ols and their analogues 38
Figure S39. Thermographs of toremifene or procyanidin B2 (**1**) (labeled as PB2) in PBST/M-PER buffer bound to the target protein EBOV-GP 39
Figure S40. Dose-response curve for the binding interaction between pCMV (negative control) and toremifene/procyanidin B2 (**1**) (PB2) 39
Table S6. Dataset overview of MST experiments for toremifene and procyanidin B2 (**1**) 39

Table S1. Preparative HPLC condition to separate compounds from *M. perlarius* fractions.

Column	Altmann Analytik Saphir 110 C18, 12 μm (300×40mm)
Mobile phase	Gradient Water (A) with MeCN(B)
Mobile phase program	For F64-B19: 14-16% (B) in 0-60 min, 16% (B) in 60-100 min<br>For F64-B20: 14% (B) in 0-30 min, 14-17% (B) in 30-75 min and 17% (B) in 75-115 min<br>F64-B1617: 15% (B) in 0-45 min, 15-20% (B) in -100 min and 17% (B) in 75-115 min
Column temperature	room temperature
Sample concentration	100 mg/ml
Injection volume	150 μl
Flow rate	20 ml/min

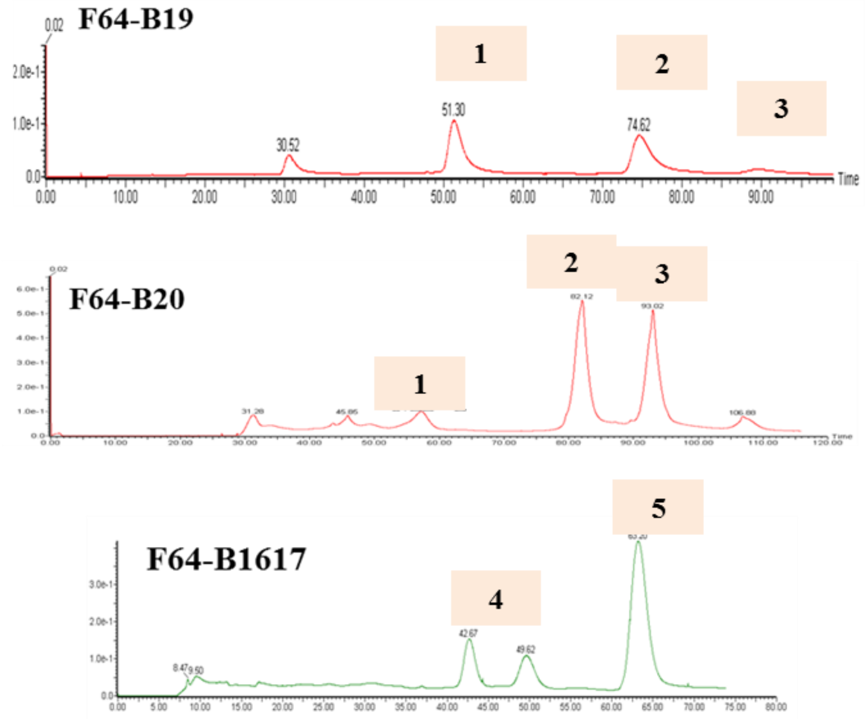

Figure S1. The chromatograms of preparative HPLC for fractions F64-B19, F64-B20 and F64-B1617 recorded at UV 210 nm.

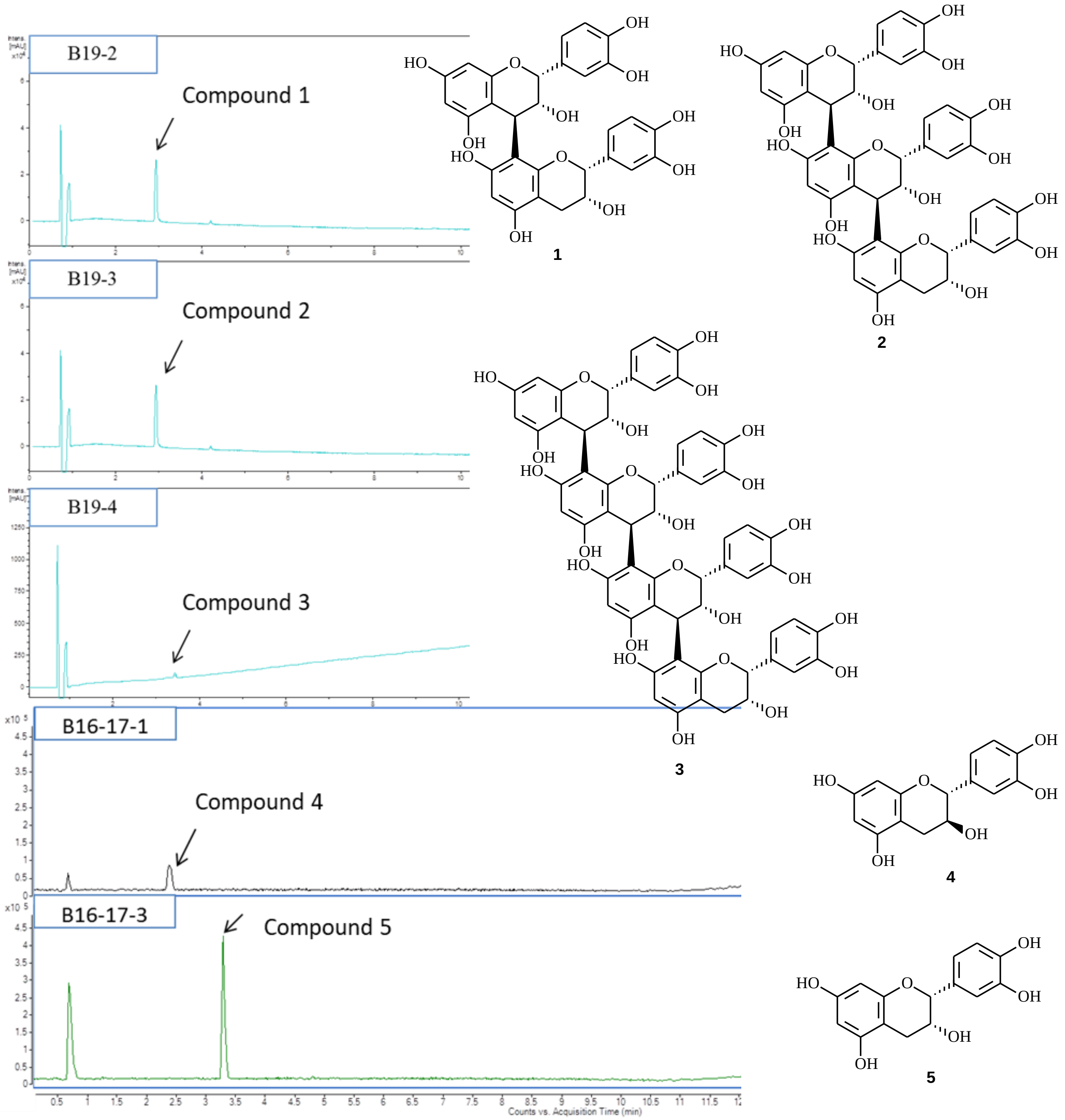

Figure S2. UPLC chromatograms of compounds **1-5** isolated from fractions B19 and B20 recorded at UV 210 nm.

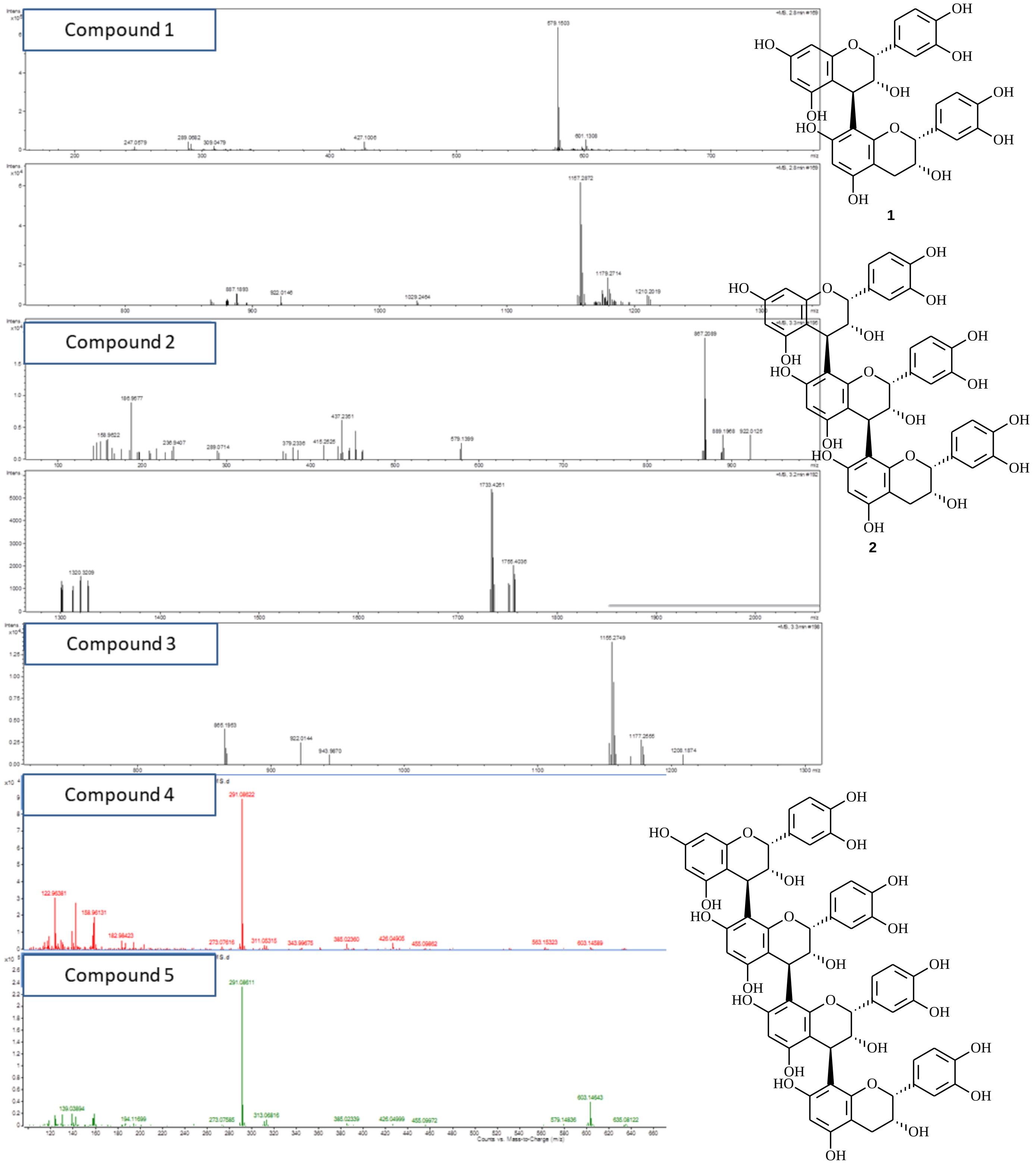

Figure S3. MS spectra of compounds **1-5**.

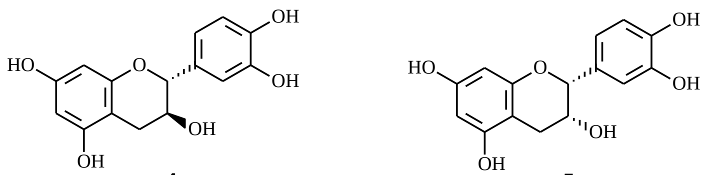

4 **5**

Table S2. Comparison of the MS data of **1-5** and their reported literature data.

	**Compound**	[M-H]$^-$ (*m/z*)	MS^2 (*m/z*)
**Experimental data**	Compound **4**	289.0721	245, 205, 179,137, 125
	Compound **5**	289.0723	245, 205, 179, 161
	Compound **1**	577.1322	425, 407, 289
	Compound **2**	865.1971	713, 575, 289
	Compound **3**	1153.2592	865, 575, 287
**Literature data**	Catechin (**4**)/ Epicatechin (**5**)	289.0702	245, 205, 179, 161, 137, 125
	Procyanidin B2 (**1**)	577.1370	451, 425, 407, 289
	Procyanidin C (**2**)	865.1	713, 575, 425, 289
	Proanthocyanidin tetramer (**3**)	1153	865, 577, 407, 423, 405

References of the literature data: catechin/epicatechin (Liu, G.-Q.; Dong, J.; Wang, H.; Wan, L.-R.; Hashi, Y.; Chen, S.-Z. ESI Ffragmentation studies of four tea catechins. *Chem. J. Chinese Univ.* **2009**, *30*, 1566–1570.), procyanidin B (Dugo, P.; Cacciola, F.; Herrero, M.; Donato, P.; Mondello, L.Use of partially porous column as second dimension in comprehensive two-dimensional system for analysis of polyphenolic antioxidants. *J. Sep. Sci.* **2008**, *31*, 3297–3308, doi:10.1002/jssc.200800281), procyanidin C (Delphine, C.; Sonia, C. Use of RP-HPLC-ESI(–)-MS/MS to differentiate various proanthocyanidin isomers in lager beer extracts. *J. Am. Soc. Brew. Chem.* **2008**, 0–6, doi:10.1094/ASBCJ-2008-0215-01) and procyanidin tetramer (Kiprovski, B.; Mikulic-Petkovsek, M.; Slatnar, A.; Veberic, R.; Stampar, F.; Malencic, D.; Latkovic, D. Comparison of phenolic profiles and antioxidant properties of European Fagopyrum esculentum cultivars. *Food Chem.* **2015**, *185*, 41–47, doi:10.1016/j.foodchem.2015.03.137).

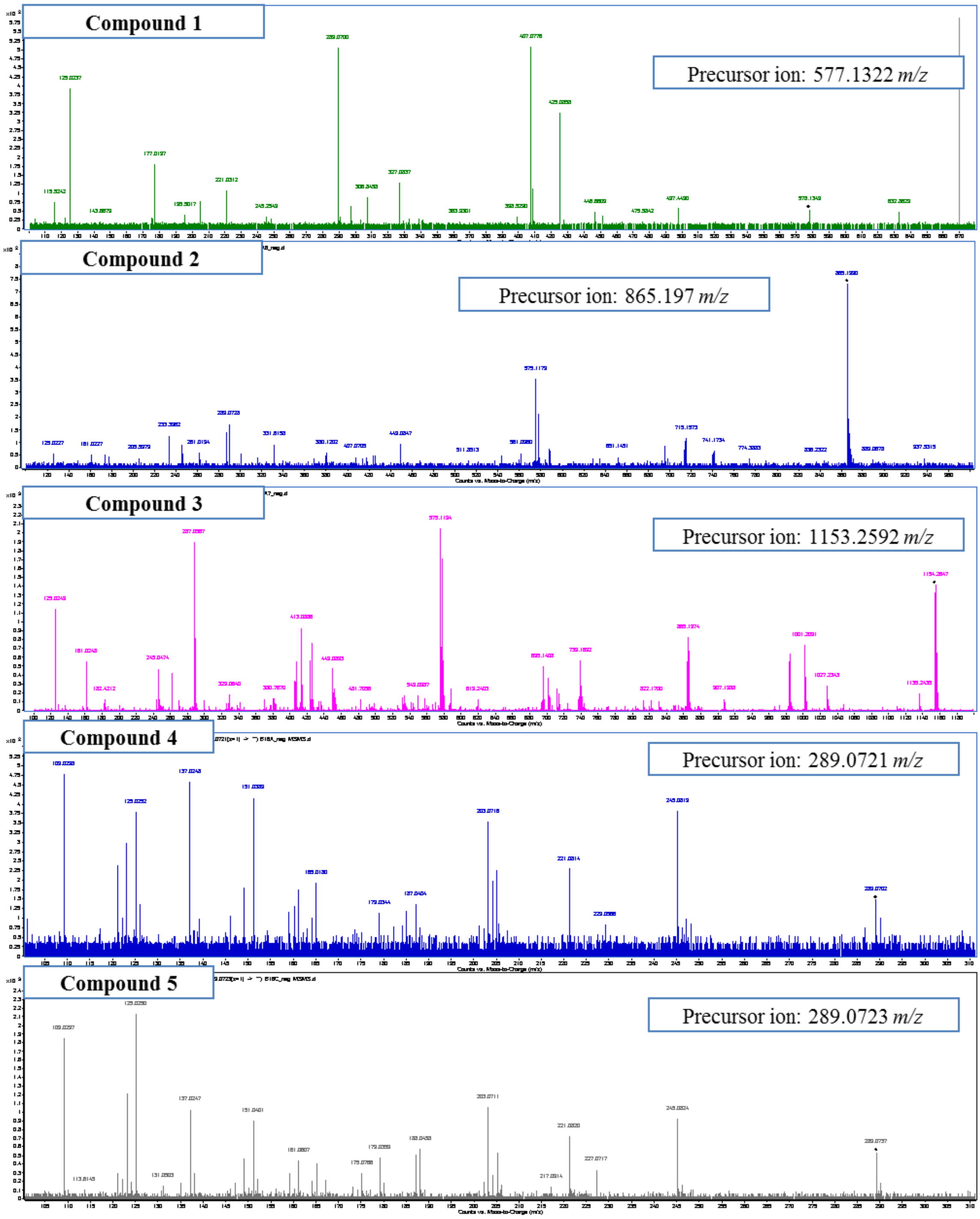

Figure S4. MS^2 spectra of **1-5**.

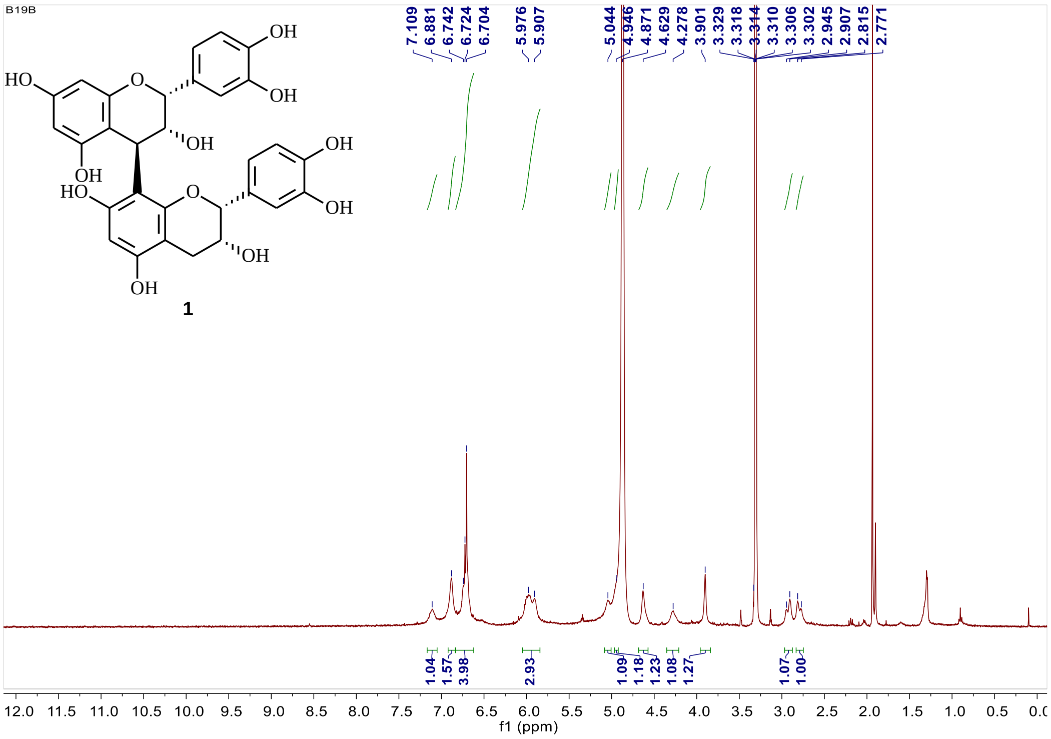

Figure S5. ^{1}H NMR spectrum of **1** in CD_3OD (400 MHz).

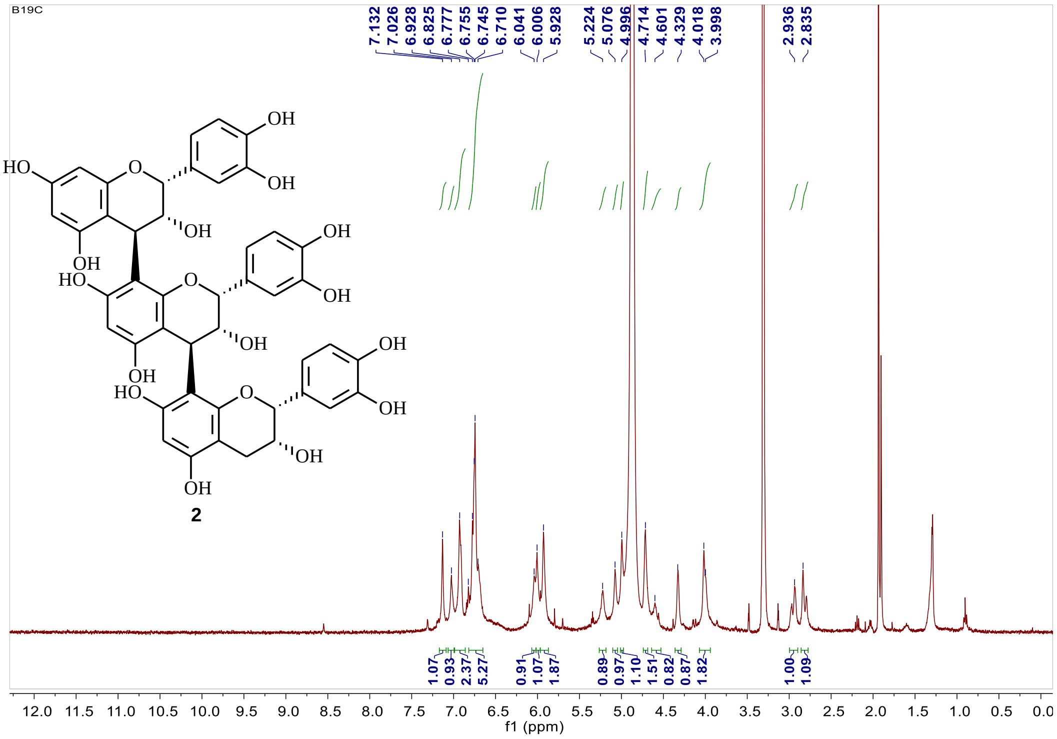

Figure S6. ^{1}H NMR spectrum of **2** in CD_3OD (400 MHz).

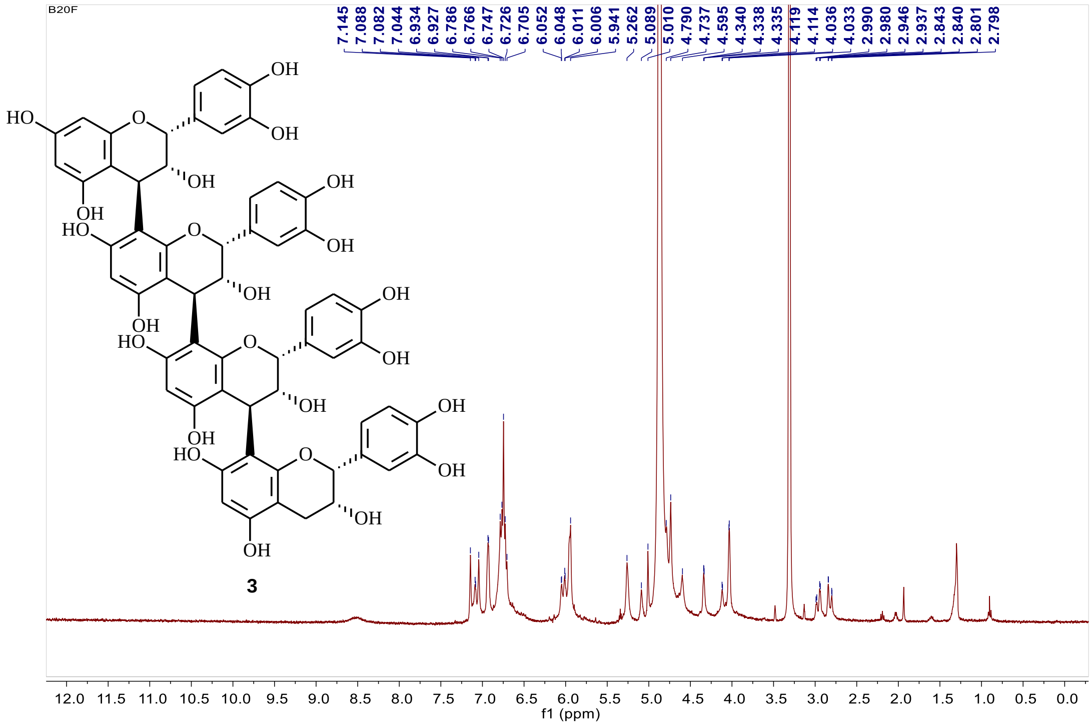

Figure S7. ^{1}H NMR spectrum of **3** in CD_3OD (400 MHz).

Table S3. Comparison of ^{1}H NMR spectral data of the literature procyanidin B2 and compound **1**.

		Procyanidin B2 (Reported by Shoji *et al*)[a]	Procyanidin B2 (Reported by Makabe *et al.*)[b]	**Compound 1**
	Position	δ_H (*J* in Hz)	δ_H (*J* in Hz)	δ_H (*J* in Hz)
A unit	2	4.95 (br s)	4.93 (m)	4.95 (brs)
	3	4.24 (br t)	4.09-4.27 (m)	4.28 (brs)
	4	2.79 (d, 17)<br>2.94 (dd, 17, 4)	2.71-2.81 (m)<br>2.84-2.93 (m)	2.81 (d, 2.9)<br>2.91 (dd, 15.5, 3.5)
	6	5.88 (s)	5.91-5.94 (m)	5.94 (brs)
	2'	7.10 (d, 2)	7.09 (br s)	7.11 (brs)
	4'	6.72 (d, 8)	6.70-6.81 (m)	6.70 (dd, 7.7, 2.2)
	5'	6.85 (dd, 8, 2)	6.70-6.81 (m)	6.73 (d, 7.7)
B unit	2	5.06 (br s)	5.05 (m)	5.04 (brs)
	3	3.79 (br s	3.91 (m)	3.90 (brs)
	4	4.61 (br s)	4.62 (br s)	4.63 (brs)
	6	5.92 (d, 2)	5.91-5.94 (m)	5.94 (brs)
	8	5.94 (d, 2)	5.91-5.94 (m)	5.94 (brs)
	2'	6.83 (d, 2)	6.89 (br s)	6.88 (d, 2.2)
	4'	6.68 (d, 8)	6.70-6.81 (m)	6.70 (dd, 7.7, 2.2)
	5'	6.60 (dd, 8, 2)	6.70-6.81 (m)	6.73 (d, 7.7)

[a]Shoji, T.; Mutsuga, M.; Nakamura, T.; Kanda, T.; Akiyama, H.; Goda, Y.Isolation and structural elucidation of some procyanidins from apple by low-temperature nuclear magnetic resonance. *J. Agric. Food Chem.* **2003**, *51*, 3806–3813, doi:10.1021/jf0300184.

[b]Makabe, H.; Kamo, T.; Hirota, M.; Mohri, Y.; Sagehashi, M.; Yamada, T.; Hattori, Y.; Morimura, K.An Efficient Synthesis of Procyanidins Using Equimolar Condensation of Catechin and/or Epicatechin Catalyzed by Ytterbium Triflate. *Heterocycles* **2009**, *79*, 549, doi:10.3987/COM-08-S(D)14.

Table S4. Comparison of ^{1}H NMR spectral data of the literature procyanidin C1 and compound **2**.

		Procyanidin PC1 (Reported by Shoji *et al.*)[a]	**2**
	Position	δ_H (*J* in Hz)	δ_H (*J* in Hz)
A unit	2	4.98 (br s)	5.00 (brs)
	3	4.31 (br s)	4.33 (s)
	4	2.81 (d, 16)<br>2.94 (dd, 4, 16)	2.83 (d, 16.0)<br>2.94 (dd, 16.0, 4.3)
	6	5.92 (s)	5.93 (s)
	2'	7.11 (d, 2)	7.13 (brs)
	4'	6.74 (d, 8)	6.71 (dd, 8.1, 2.1)
	5'	6.88	6.93 (d, 8.1)
B unit	2	5.23 (br s)	5.22 (brs)
	3	3.94 (br s)	4.01 (d, 8.1)
	4	4.68 (br s)	4.71 (d, 8.8)
	6	5.88 (s)	5.93 (s)
	2'	7.02 (d, 8)	7.03 (brs)
	4'	6.71	6.77 (dd, 8.1, 2.0)
	5'	6.67 (d, 2)	6.74 (d, 8.0)
C unit	2	5.06 (br s)	5.08 (brs)
	3	3.97 (br s)	4.01 (d, 8.1)
	4	4.68 (br s)	4.60 (brs)
	6	5.97 (d, 2)	6.01 (d, 1.9)
	8	6.00 (d, 2)	6.04 (brs)
	2'	6.89 (d, 2)	6.92 (d, 2.0 Hz)
	4'	6.72	6.77 (dd, 8.1, 2.0)
	5'	6.69	6.74 (d, 8.0)

[a]Shoji, T.; Mutsuga, M.; Nakamura, T.; Kanda, T.; Akiyama, H.; Goda, Y.Isolation and structural elucidation of some procyanidins from apple by low-temperature nuclear magnetic resonance. *J. Agric. Food Chem.* **2003**, *51*, 3806–3813, doi:10.1021/jf0300184.

Table S5. Comparison of ^{1}H NMR spectral data of the literature procyanidin tetramer epicatechin-(4β→8)-epicatechin-(4β→8)-epicatechin-(4 β→8)-epicatechin and compound **3**.

		Procyanidin Tetramer 11 (Reported by Shoji *et al.*)[a]	**3**
	Position	δ_H (*J* in Hz)	δ_H (*J* in Hz)
A unit	2	5.00 (br s)	5.01 (brs)
	3	4.32 (br t)	4.34 (brs)
	4	2.81 (d, 17)<br>2.96 (dd, 4, 17)	2.84 (dd, 17.0<br>2.95 (dd, 17.0, 3.7)
	6	5.94 (s)	5.94 (brs)
	2'	7.13 (d, 2)	7.14 (brs)
	4'	6.75 (d, 8)	6.78 (dd, 8.3, 2.3)
	5'	6.91	6.93 (brs)
B unit	2	5.29 (br s)	5.26 (brs)
	3	3.97 (d, 2)	4.03 (brs)
	4	4.71 (br s)	4.74 (brs)
	6	5.95 (s)	5.94 (brs)
	2'	7.03 (d, 2)	7.04 (brs)
	4'	6.69 (d, 8)	6.74 (d, 8.3)
	5'	6.75	6.74 (d, 8.3)
C unit	2	5.26 (br s)	5.26 (brs)
	3	4.08 (d, 2)	4.12 (brs)
	4	4.75 (br s)	4.74 (brs)
	6	5.93 (s)	5.94 (brs)
	2'	7.09 (d, 2)	7.09 (brs)
	4'	6.73 (d, 8)	6.78 (dd, 8.3, 2.3)
	5'	6.78	6.74 (d, 8.3)
D unit	2	5.09 (br s)	5.09 (brs)
	3	3.99 (d, 2)	4.03 (brs)
	4	4.72 (br s)	4.79 (brs)
	6	5.98 (d, 2)	6.01 (brs)
	8	6.01 (d, 2)	6.05 (brs)
	2'	6.91 (d, 2)	6.93 (brs)
	4'	6.74 (d, 8)	6.78 (dd, 8.3, 2.3)
	5'	6.70	6.71 (d, 8.3)

[a]Shoji, T.; Mutsuga, M.; Nakamura, T.; Kanda, T.; Akiyama, H.; Goda, Y.Isolation and structural elucidation of some procyanidins from apple by low-temperature nuclear magnetic resonance. *J. Agric. Food Chem.* **2003**, *51*, 3806–3813, doi:10.1021/jf0300184.

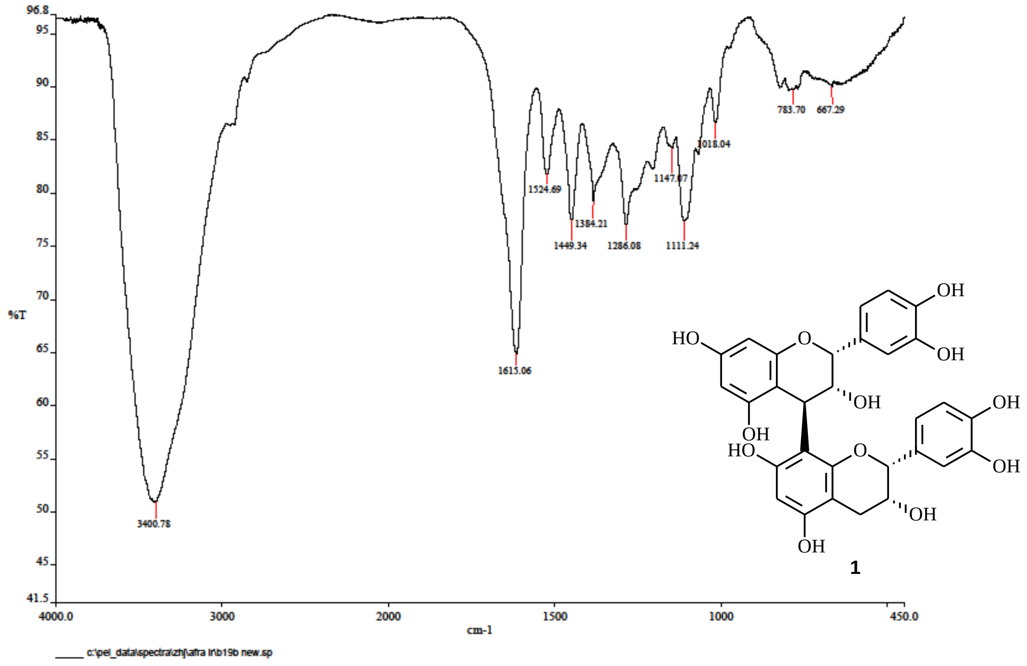

Figure S8. IR spectrum of **1**.

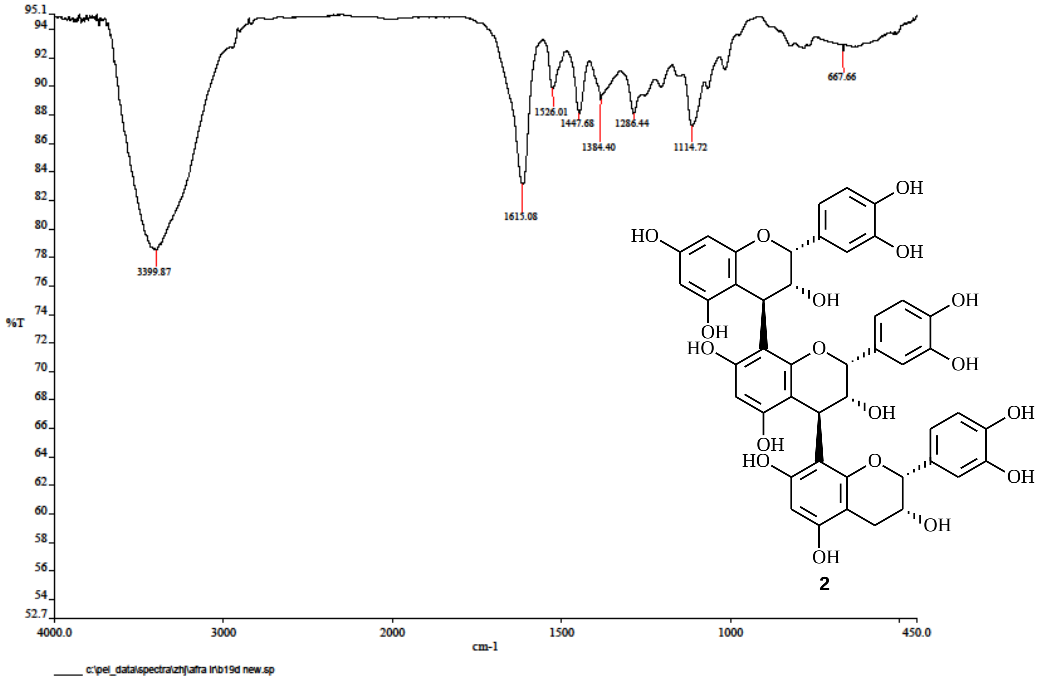

Figure S9. IR spectrum of **2**.

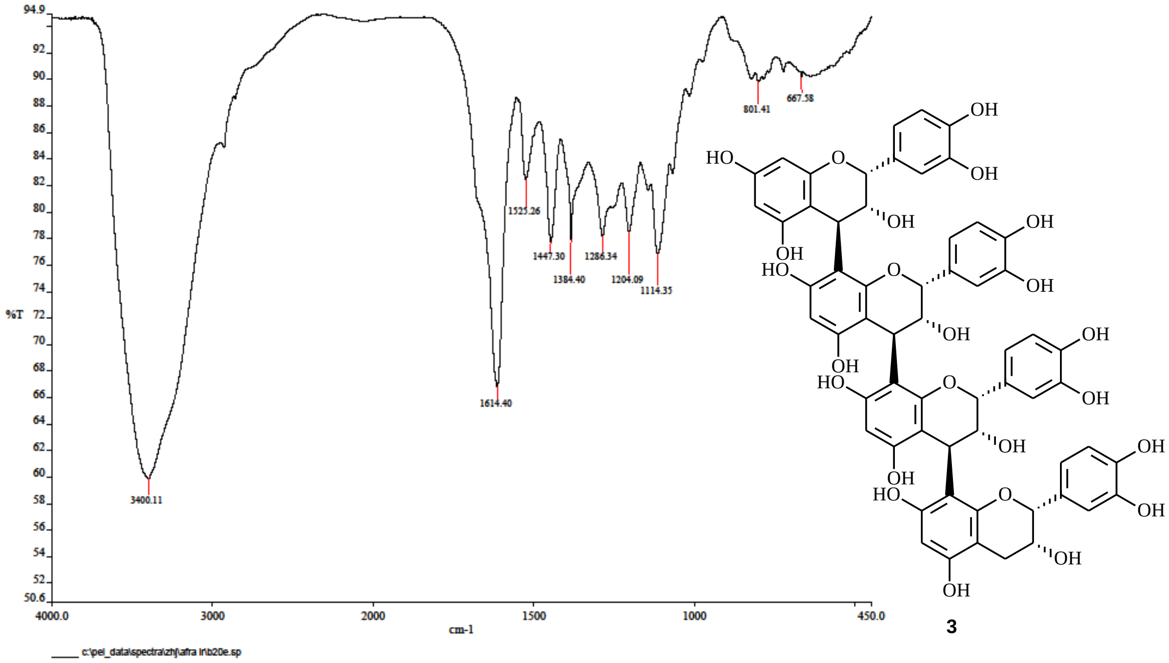

Figure S10. IR spectrum of **3**.

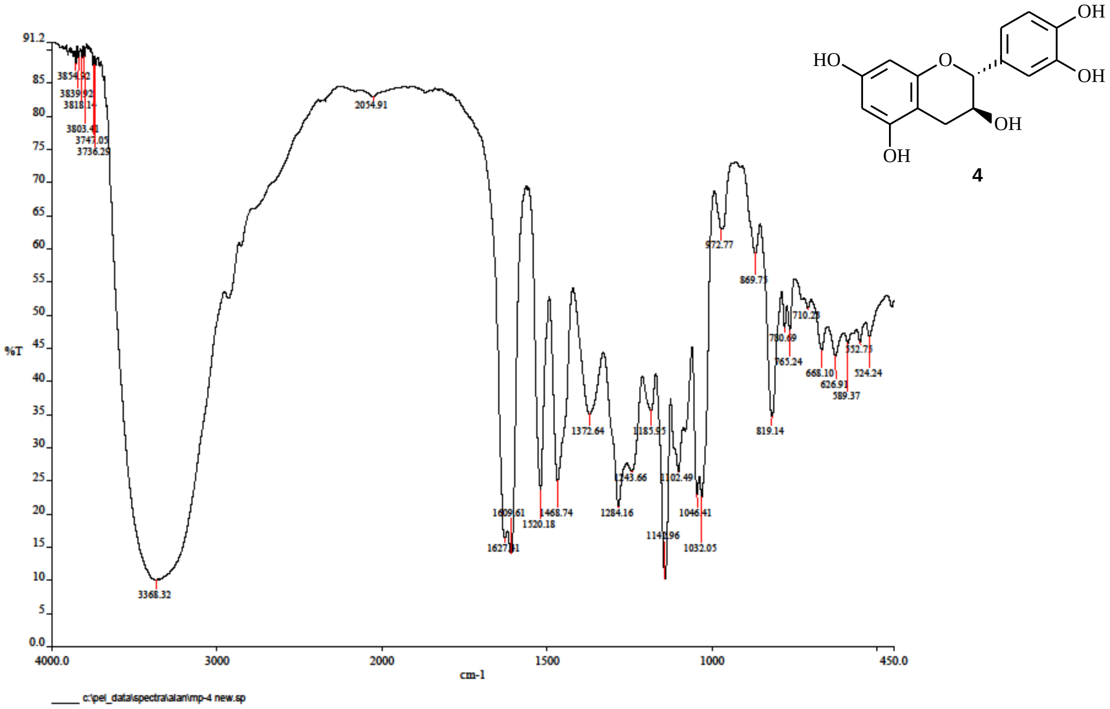

Figure S11. IR spectrum of **4**.

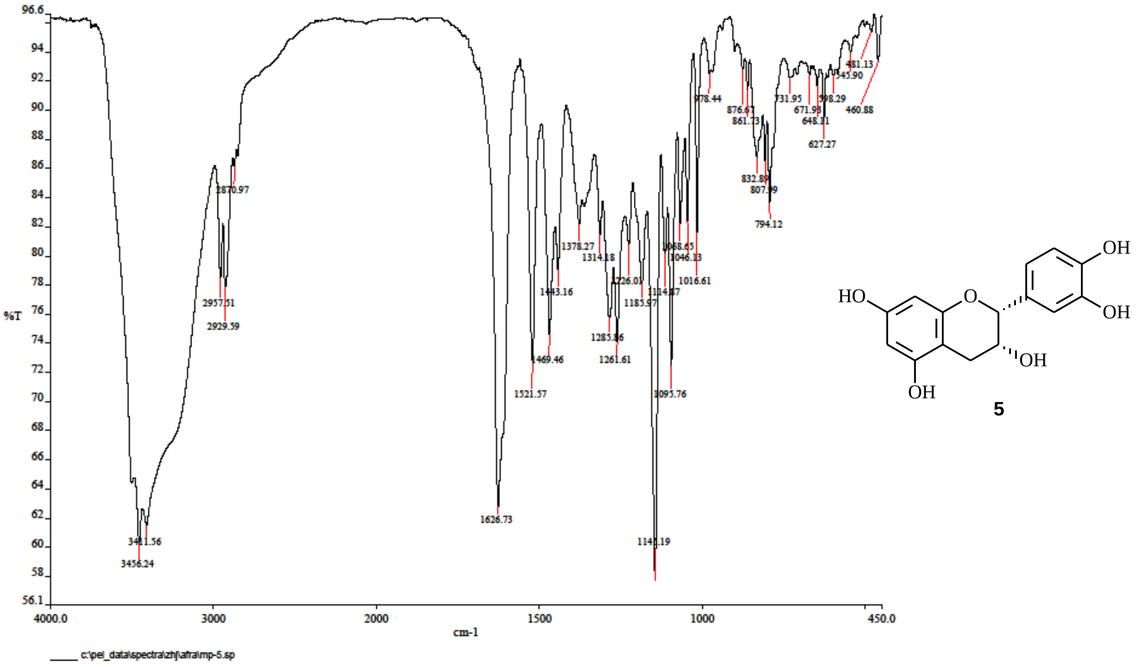

Figure S12. IR spectrum of **5**.

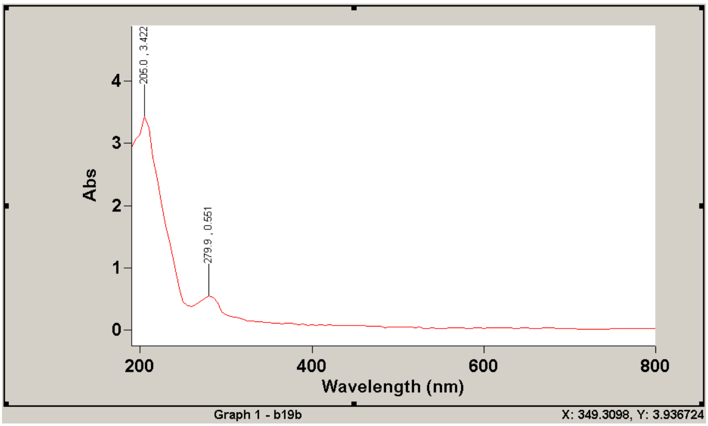

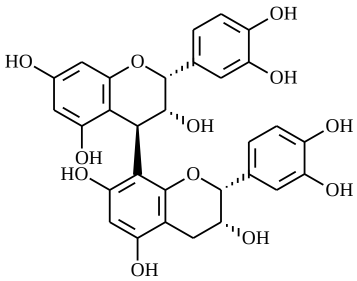

1

Figure S13. UV spectrum of **1**.

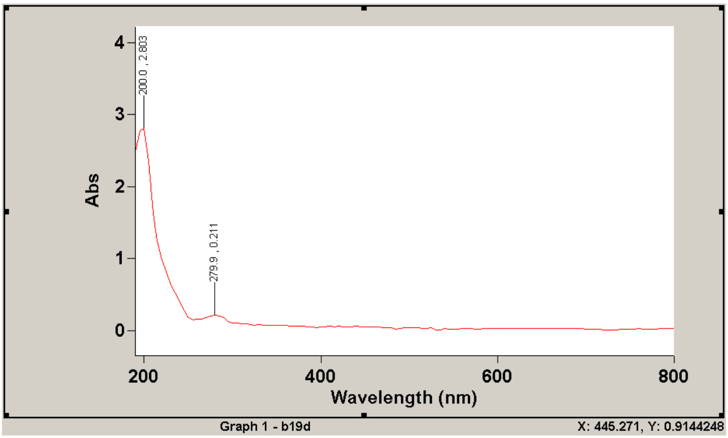

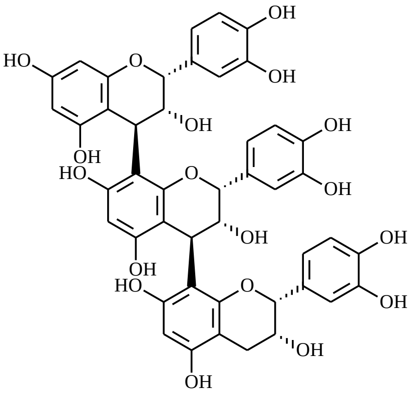

2

Figure S14. UV spectrum of **2**.

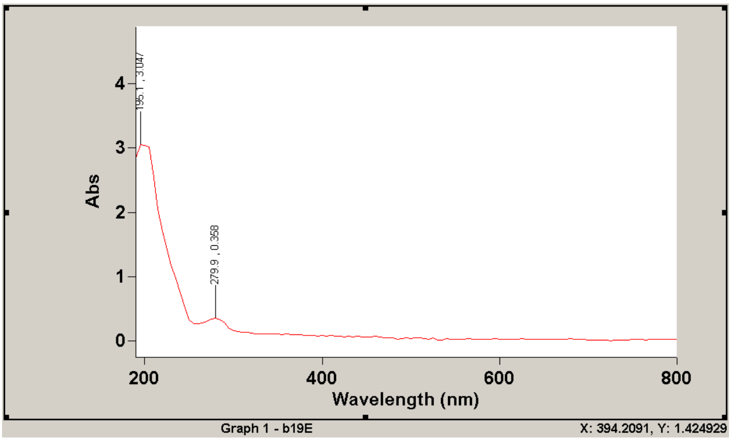

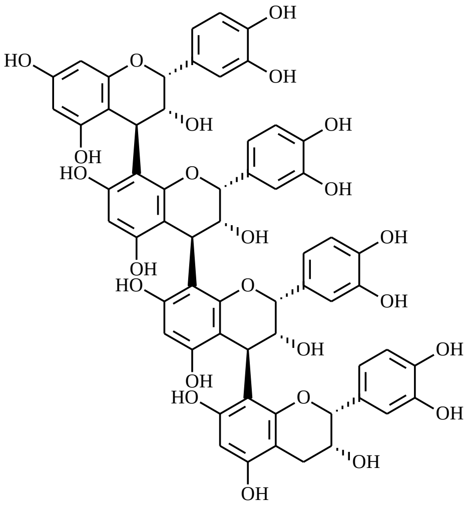

Figure S15. UV spectrum of **3**.

3

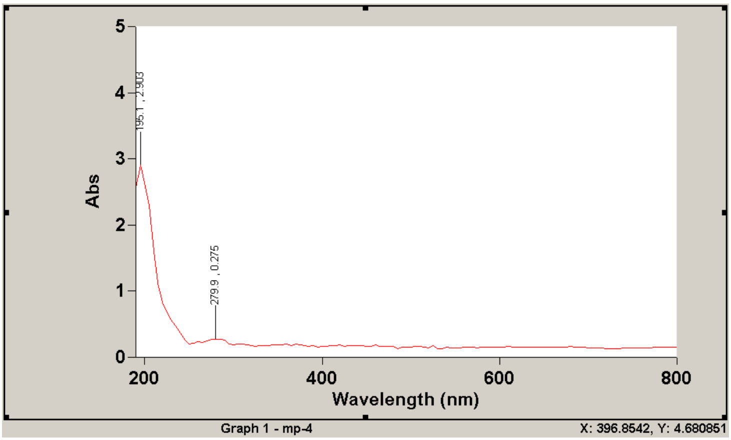

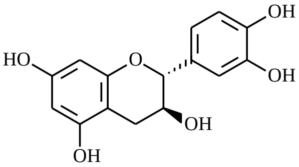

4

Figure S16. UV spectrum of **4**.

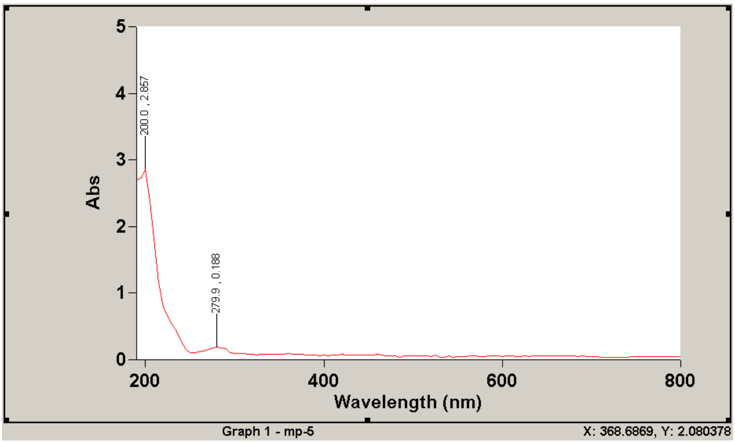

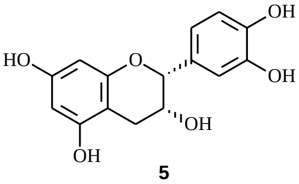

5

Figure S17. UV spectrum of **5**.

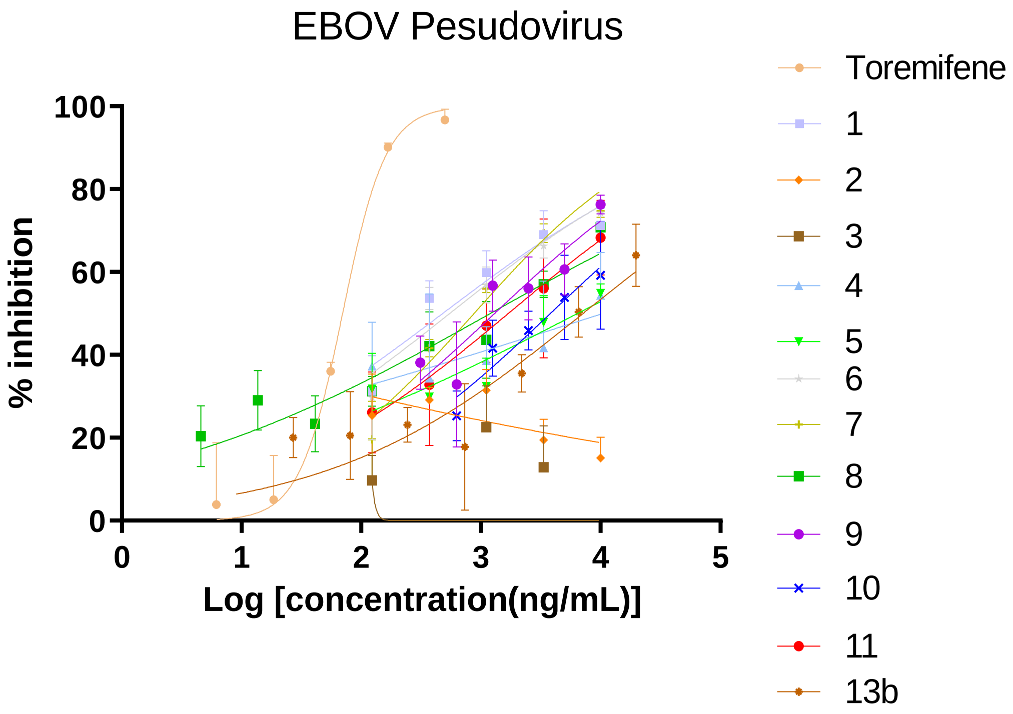

Figure S18. *In vitro* dose-response curves of plant extract fractions, flavan-3-ols and their derivatives on pseudovirion HIV-Luc/EBOV-GP. Samples were evaluated in A549 cells against pseudovirion HIV-Luc/EBOV-GP. The IC_{50} values were calculated by four-parameter dose-response curve-fitting in GraphPad.

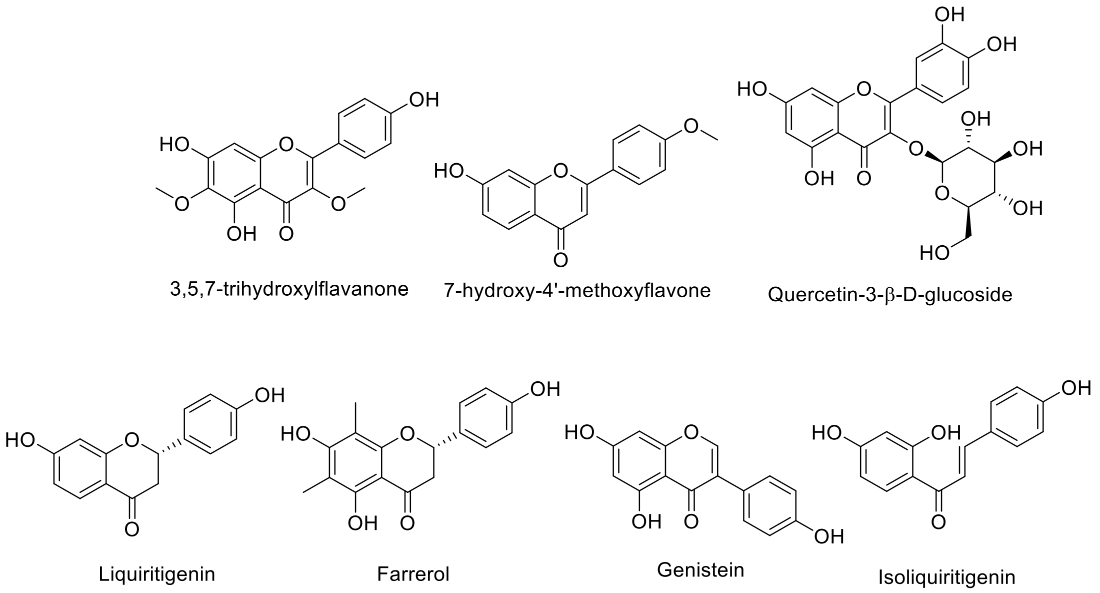

Figure S19. Chemical structures of flavanones showing no inhibitory effects against EBOV pseudovirus at concentrations of 50 μM.

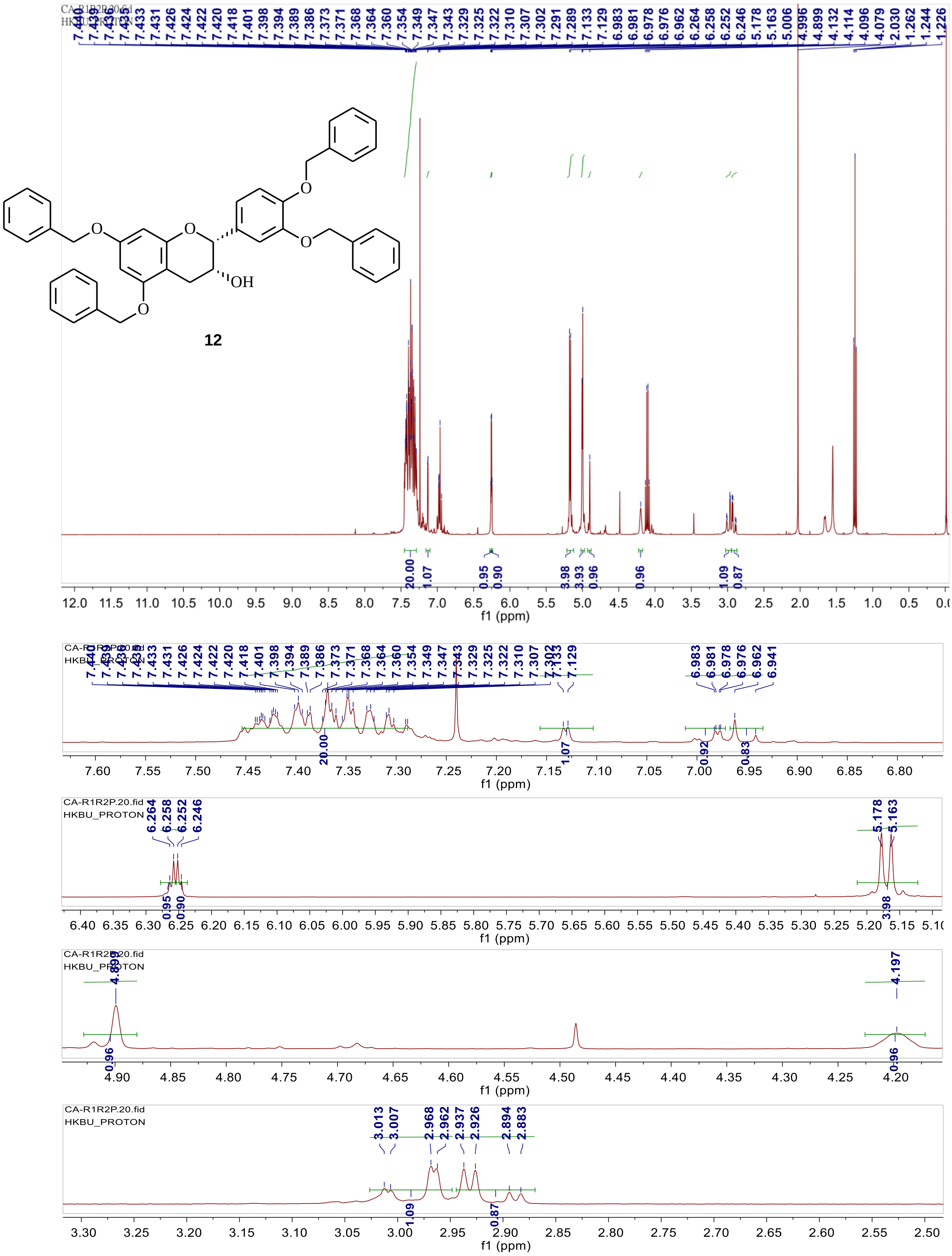

Figure S20. ^{1}H NMR spectrum of **12** in $CDCl_3$ (400 MHz).

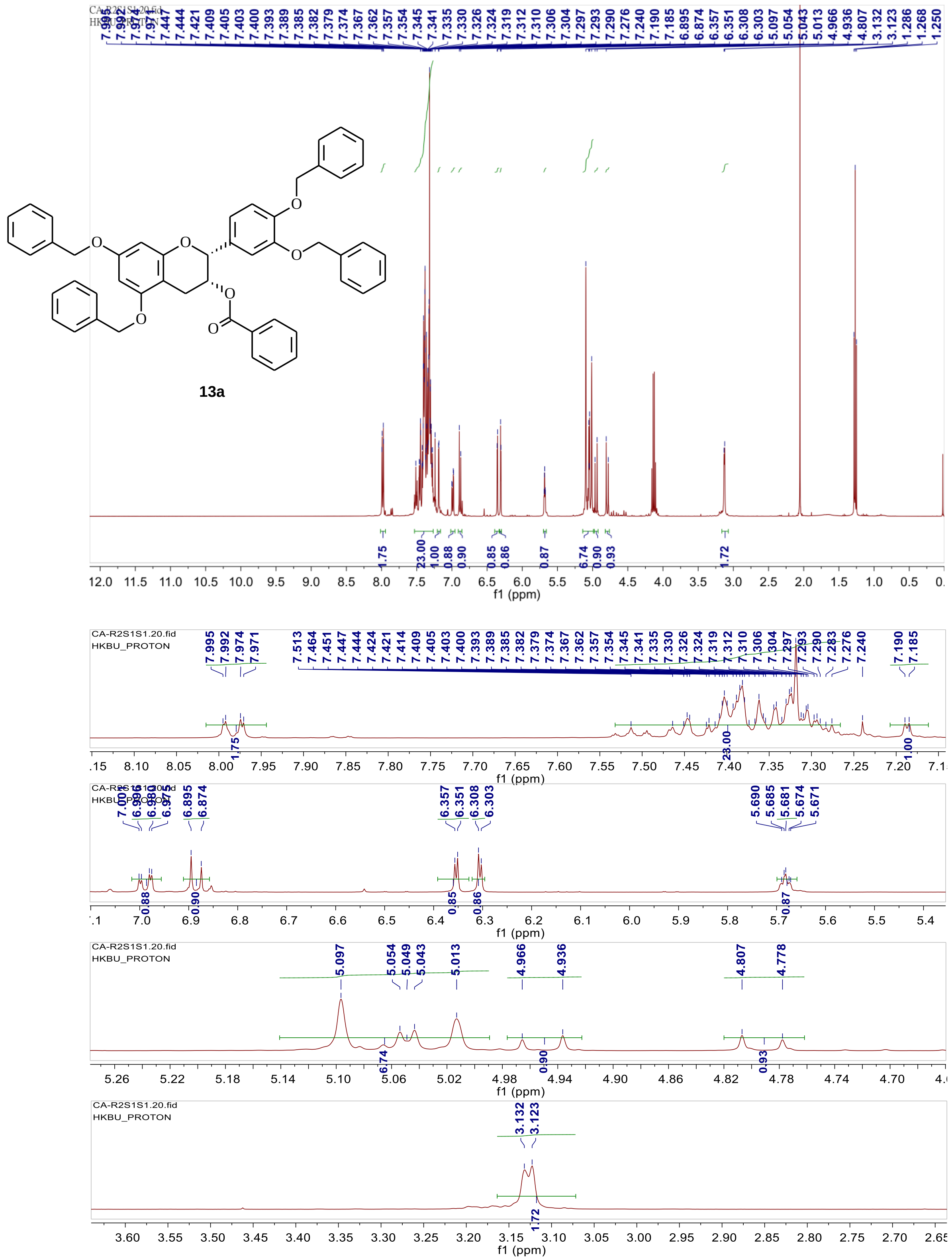

Figure S21. ^{1}H NMR spectrum of **13a** in $CDCl_3$ (400 MHz).

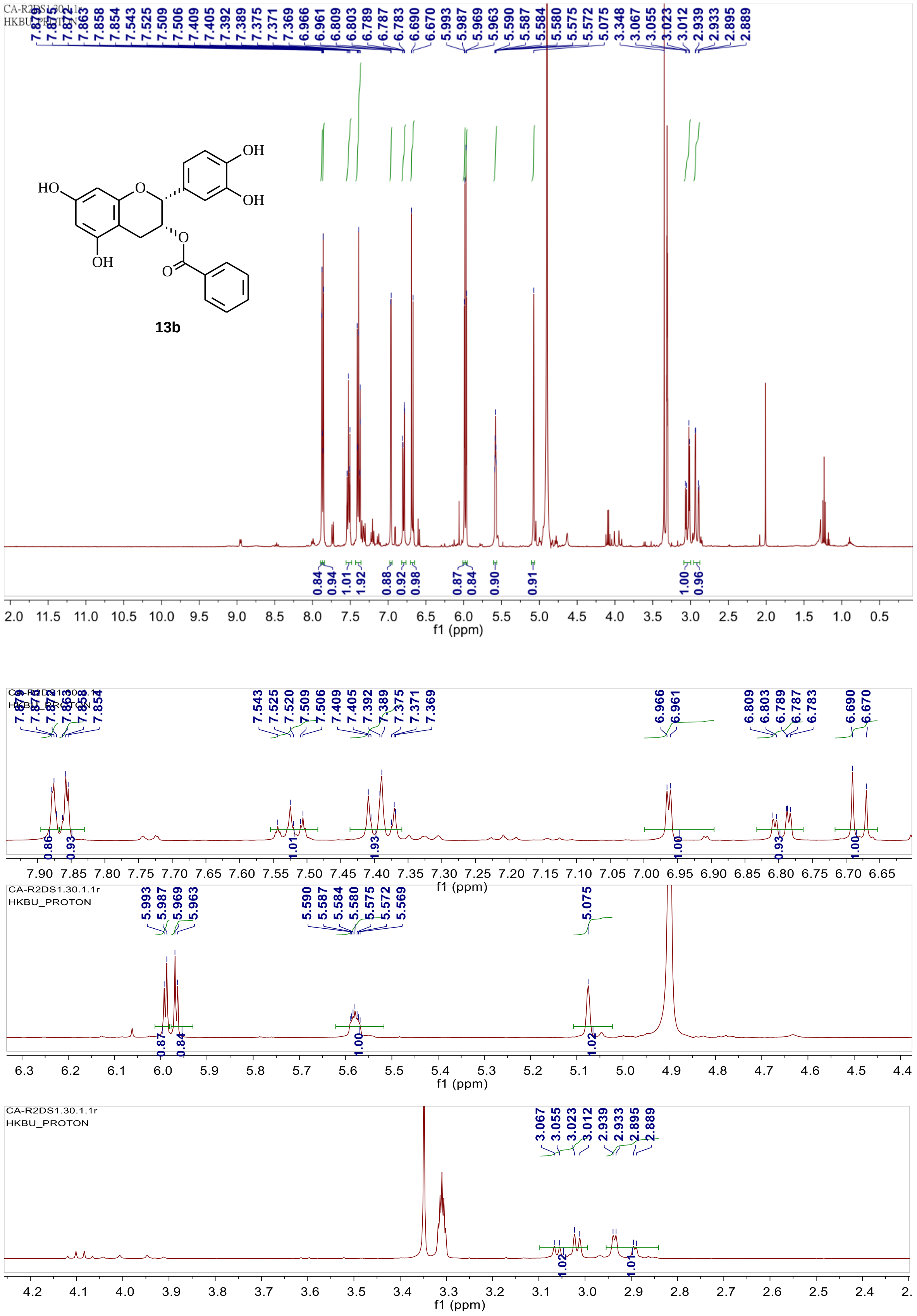

Figure S22. ^{1}H NMR spectrum of **13b** in CD_3OD (400 MHz).

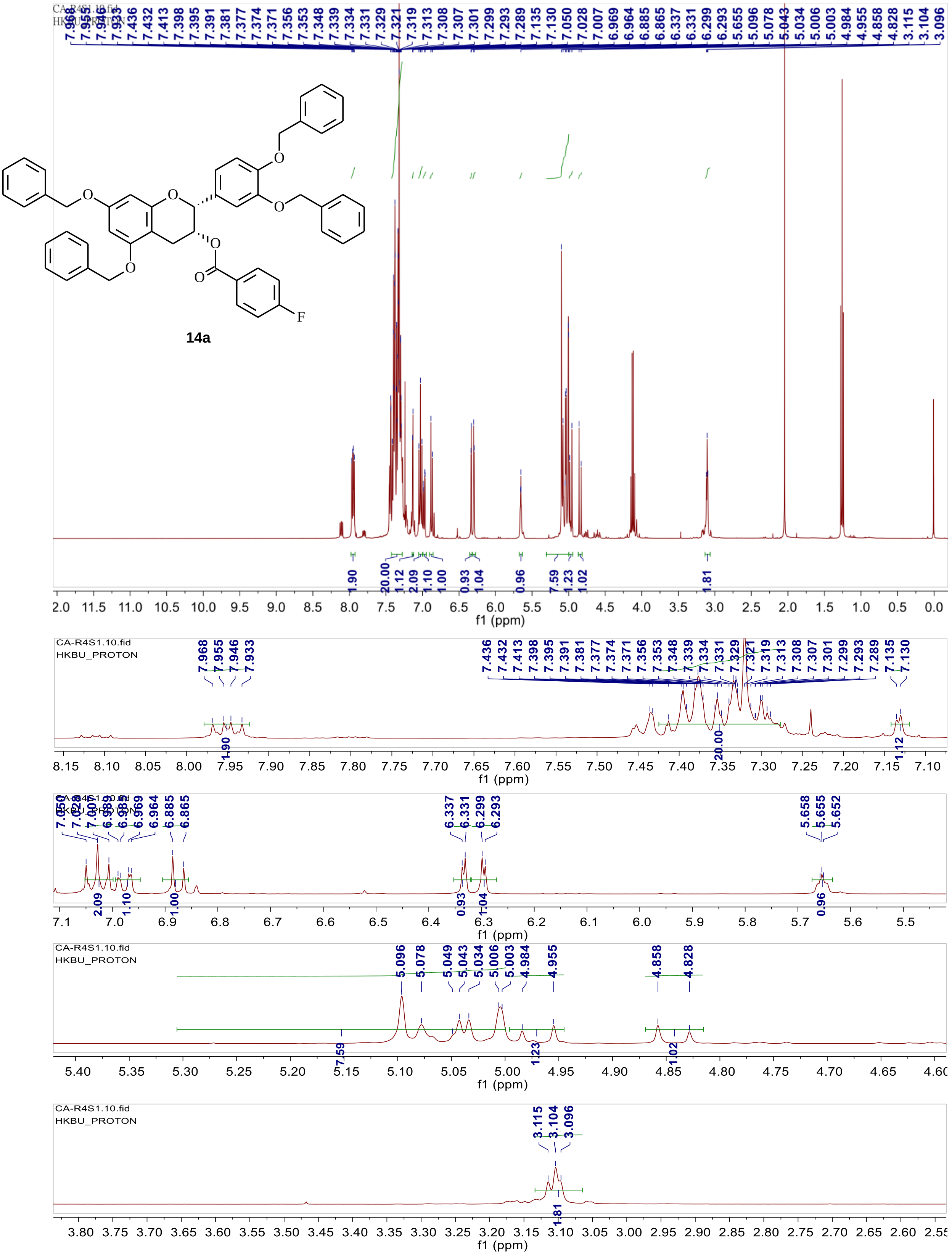

Figure S23. ^{1}H NMR spectrum of **14a** in $CDCl_3$ (400 MHz).

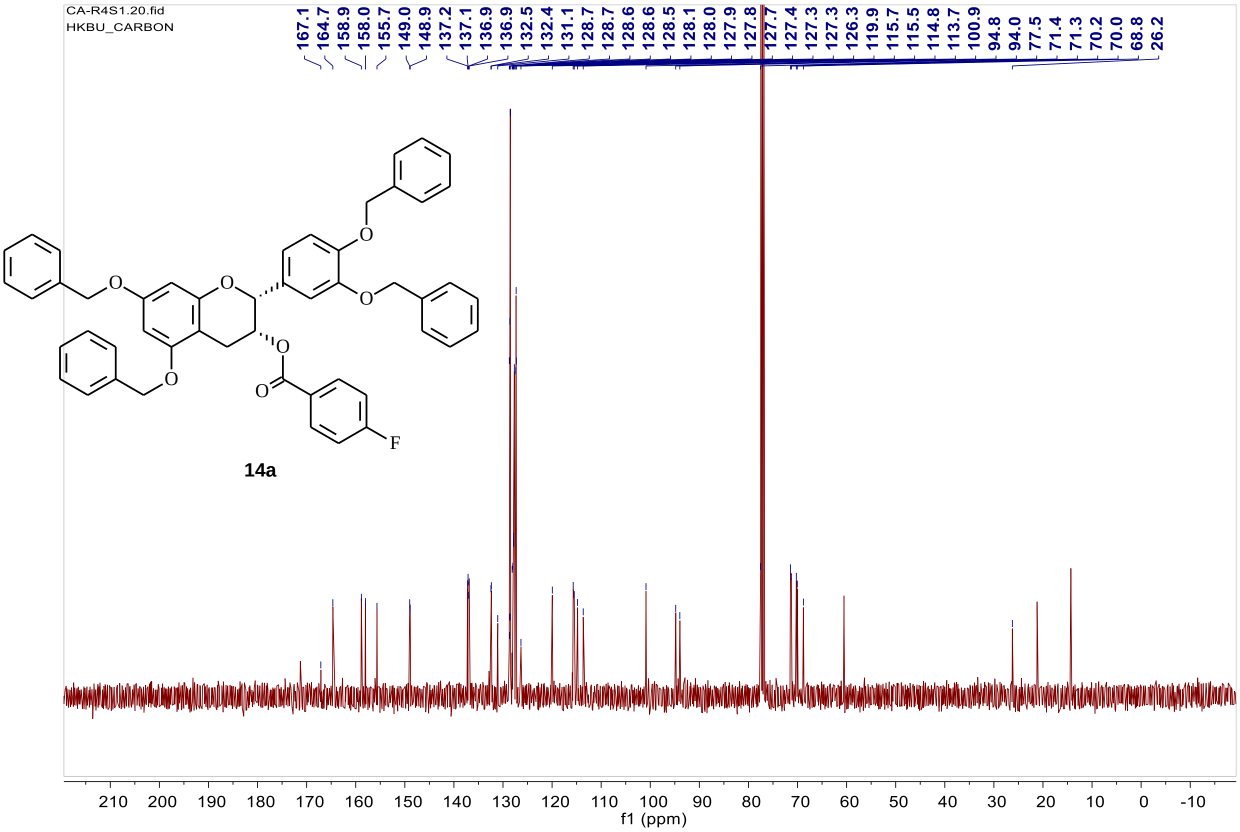

Figure S24. ^{13}C NMR spectrum of **14a** in $CDCl_3$ (100 MHz).

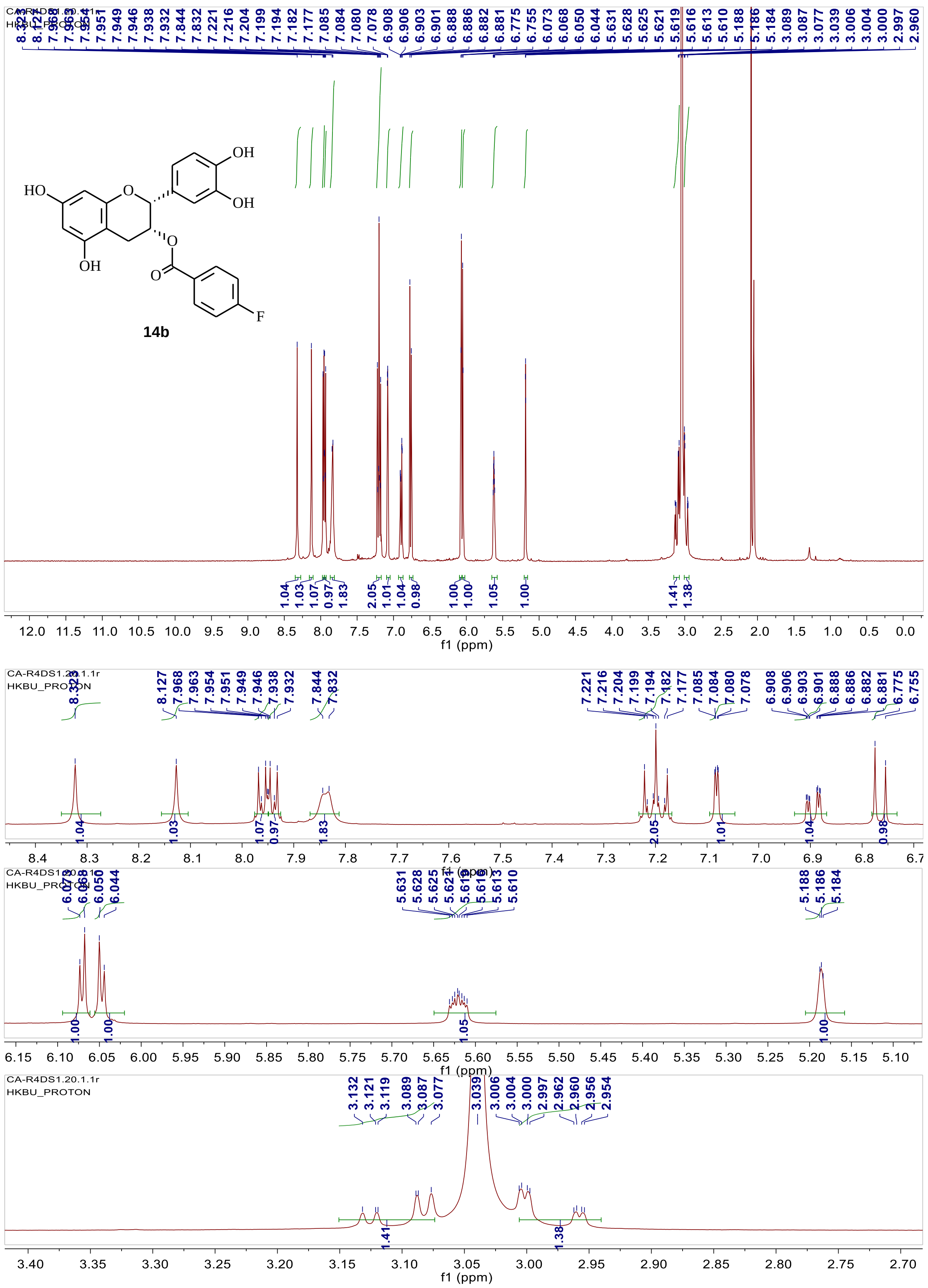

Figure S25. ^{1}H NMR spectrum of **14b** in acetone-d_6 (400 MHz).

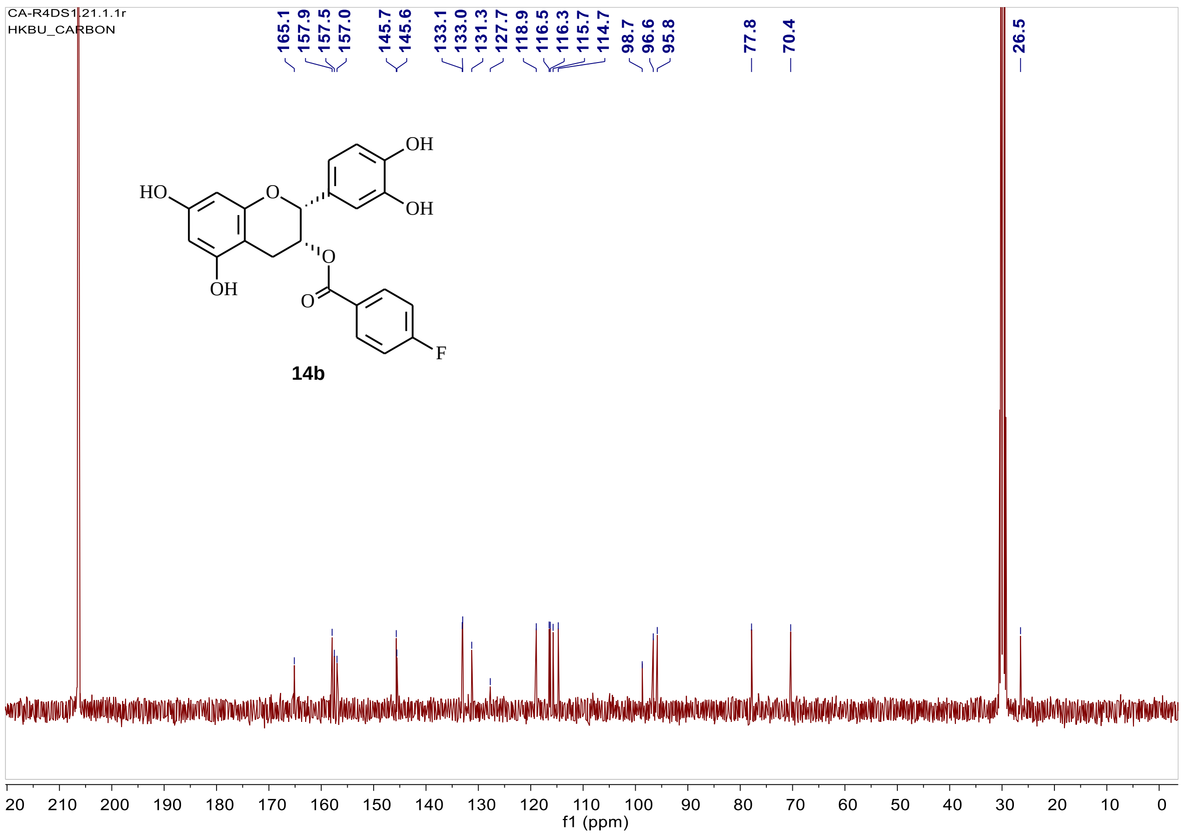

Figure S26. ^{13}C NMR spectrum of **14b** in acetone-d$_6$ (100 MHz).

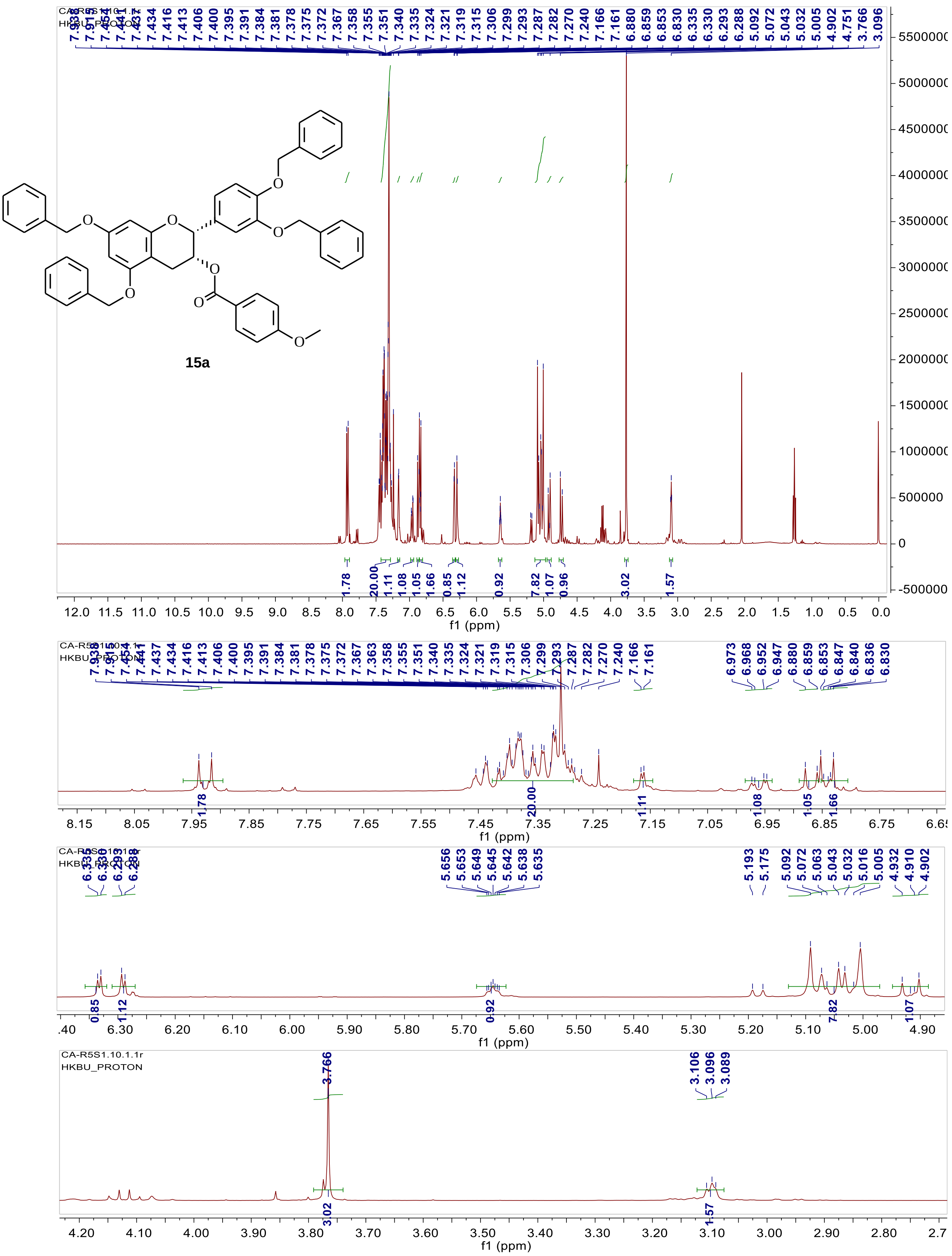

Figure S27. ^{1}H NMR spectrum of **15a** in $CDCl_3$ (400 MHz).

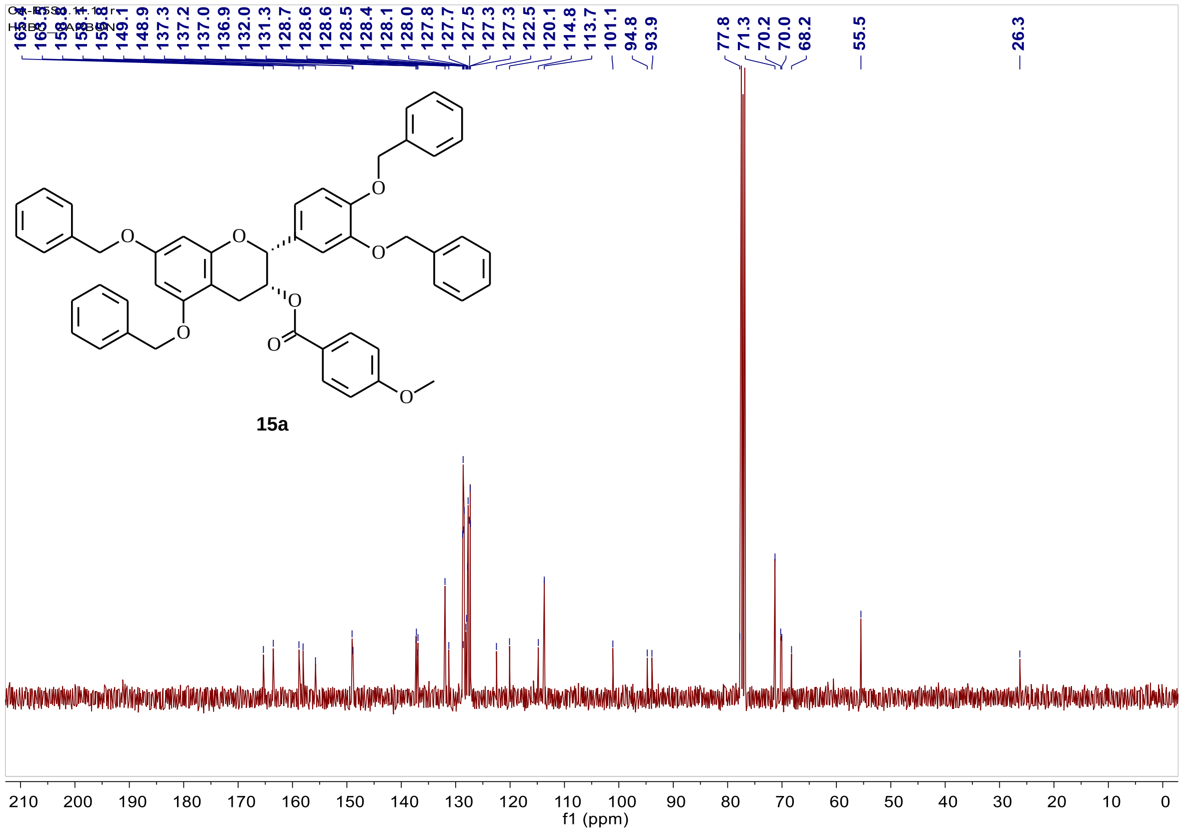

Figure S28. ^{13}C NMR spectrum of **15a** in $CDCl_3$ (100 MHz).

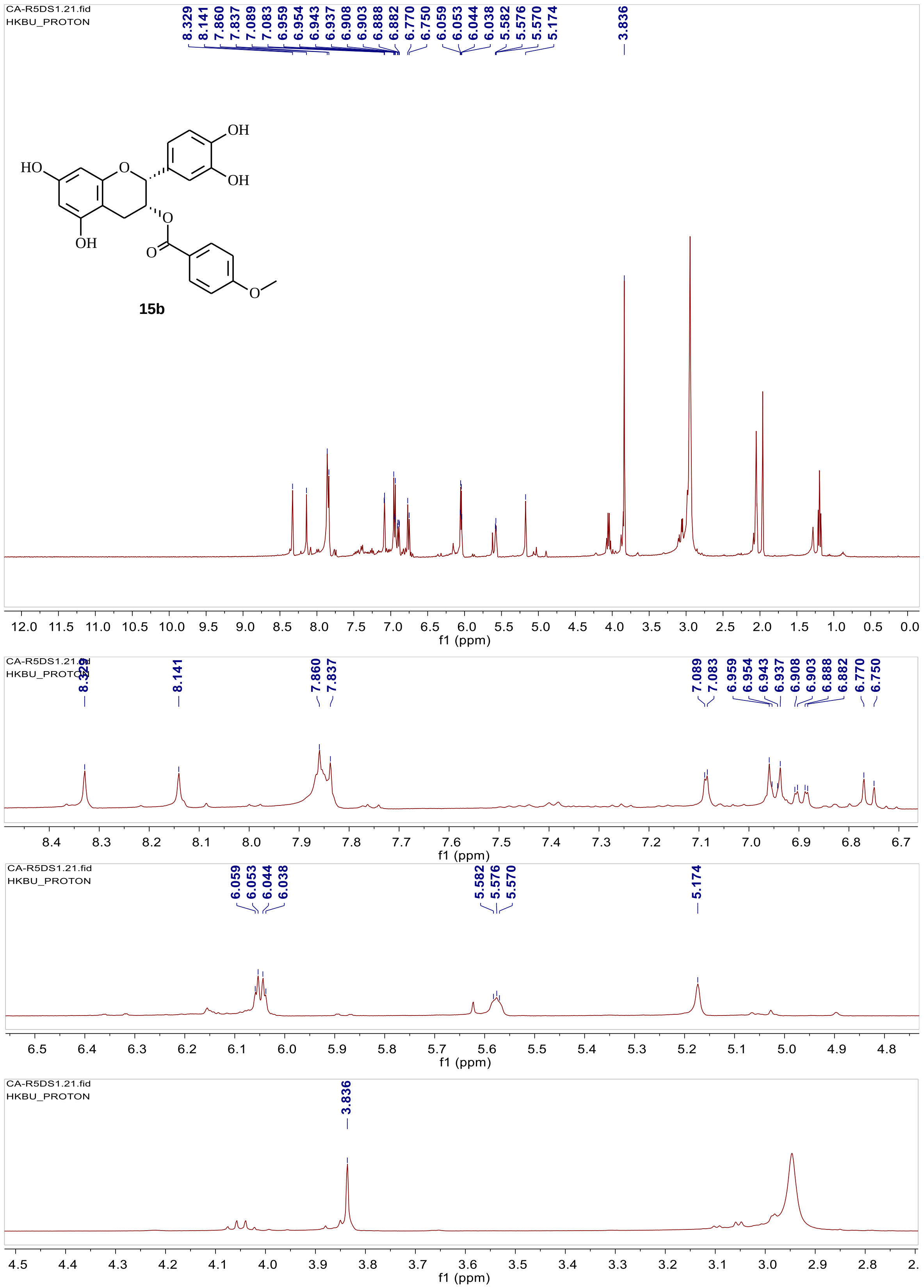

Figure S29. ^{1}H NMR spectrum of **15b** in acetone-d_6 (400 MHz).

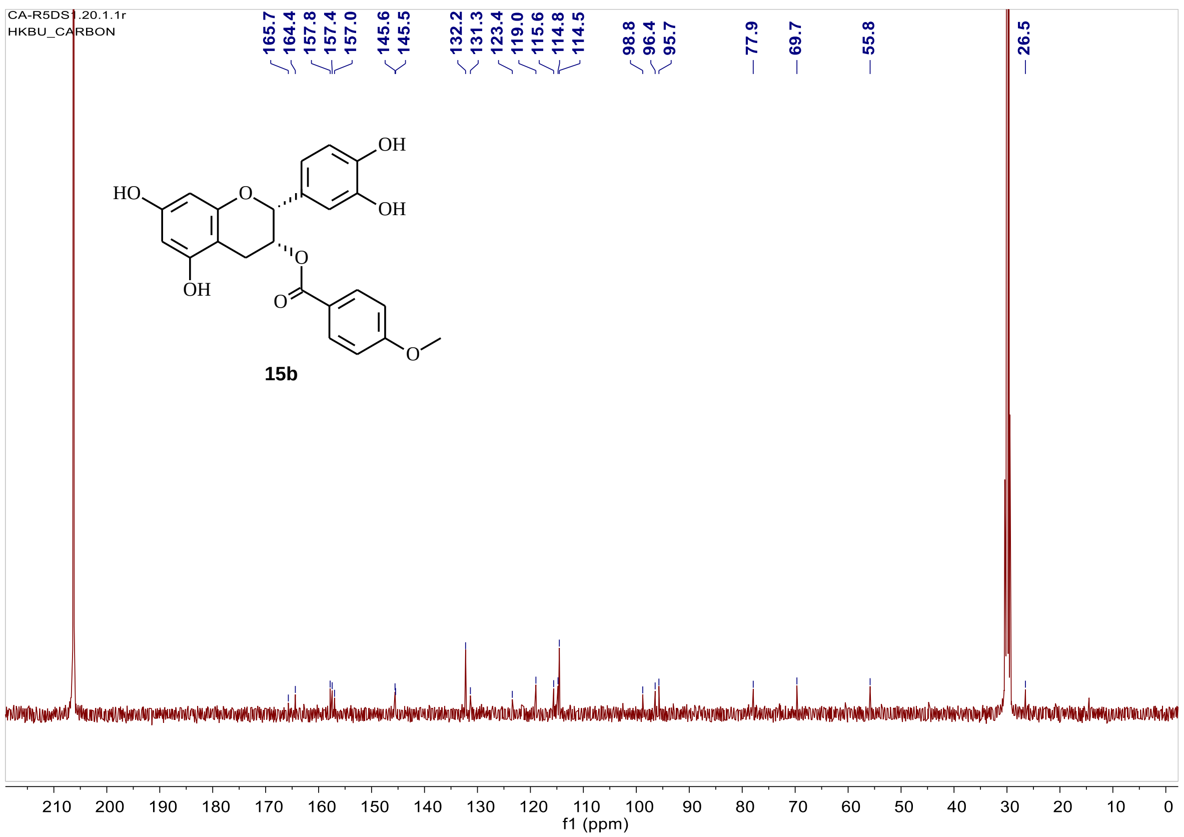

Figure S30. ^{13}C NMR spectrum of **15b** in acetone-d6 (100 MHz).

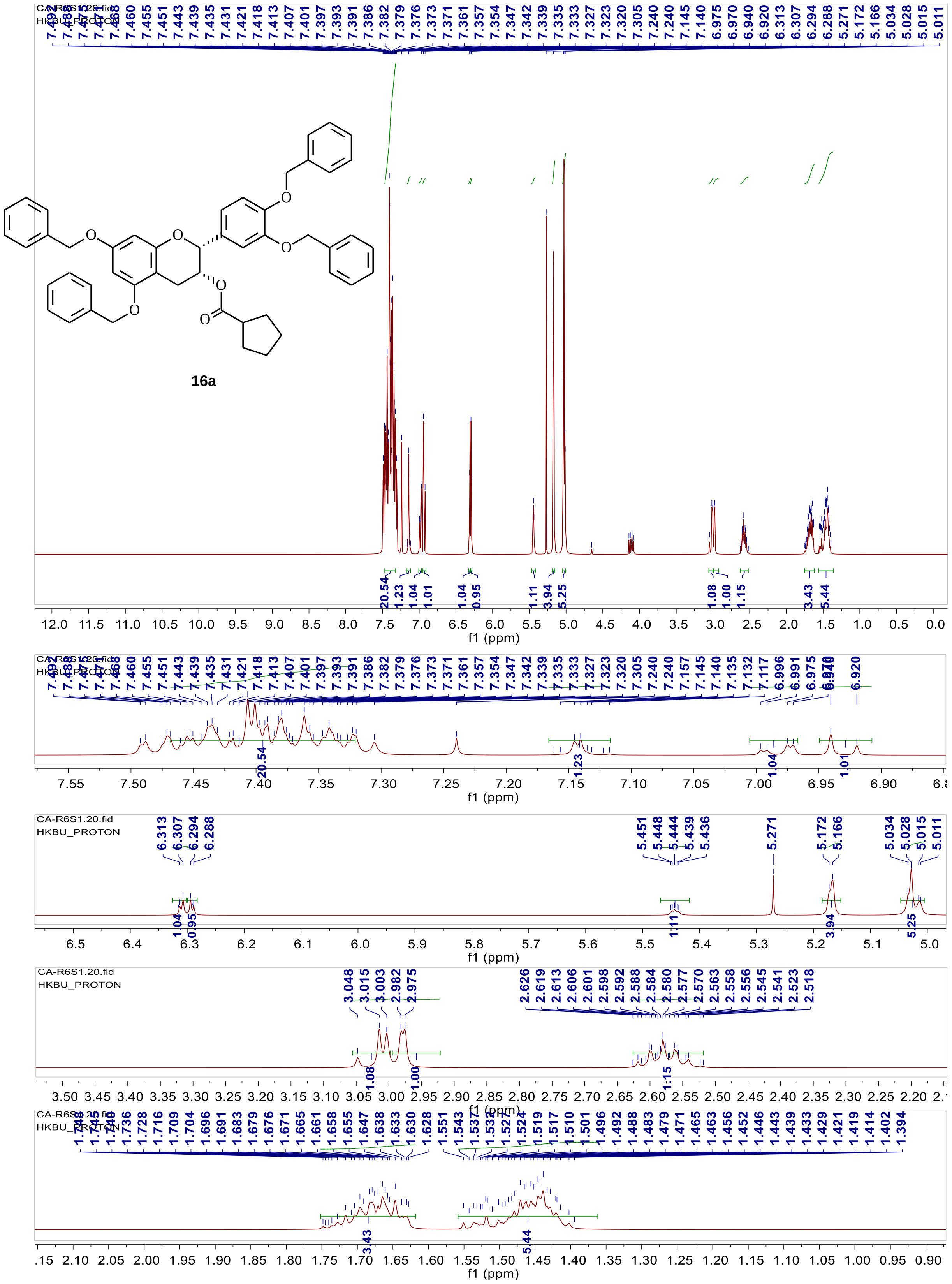

Figure S31. ^{1}H NMR spectrum of **16a** in $CDCl_3$ (400 MHz).

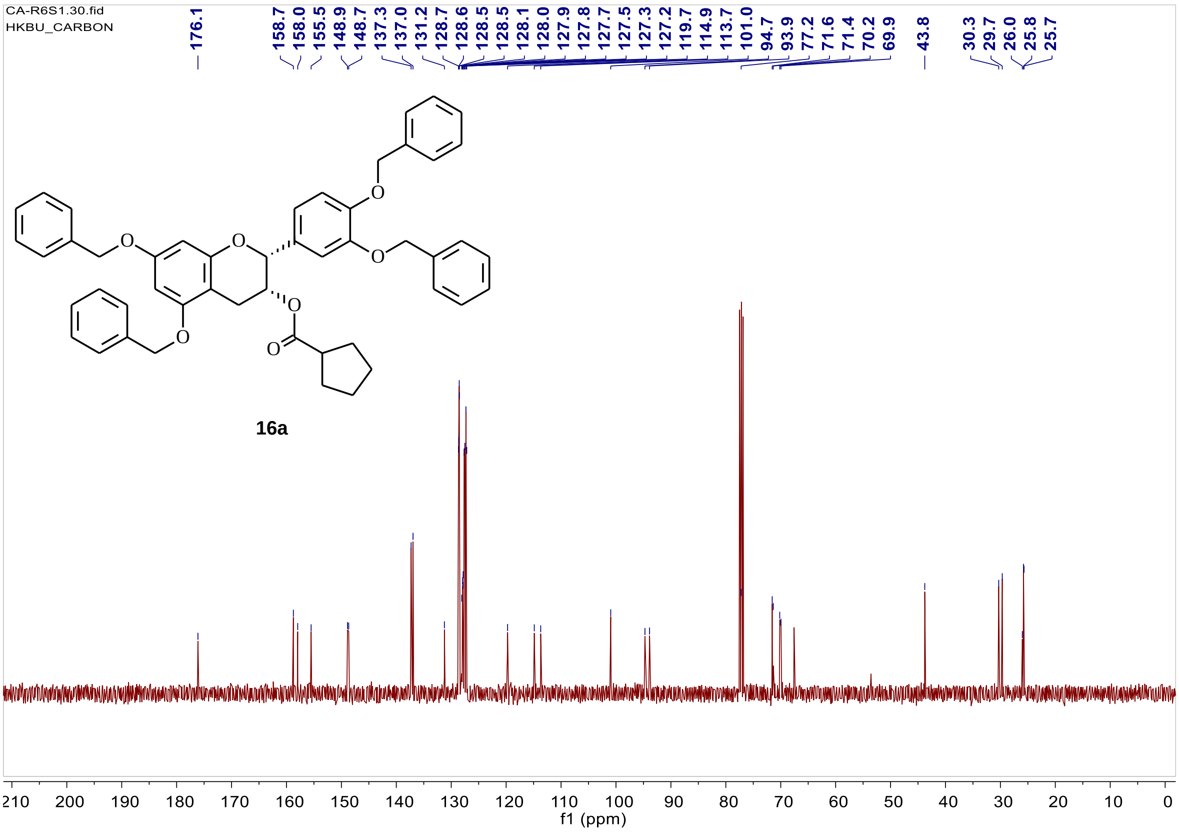

Figure S32. ^{13}C NMR spectrum of **16a** in $CDCl_3$ (100 MHz).

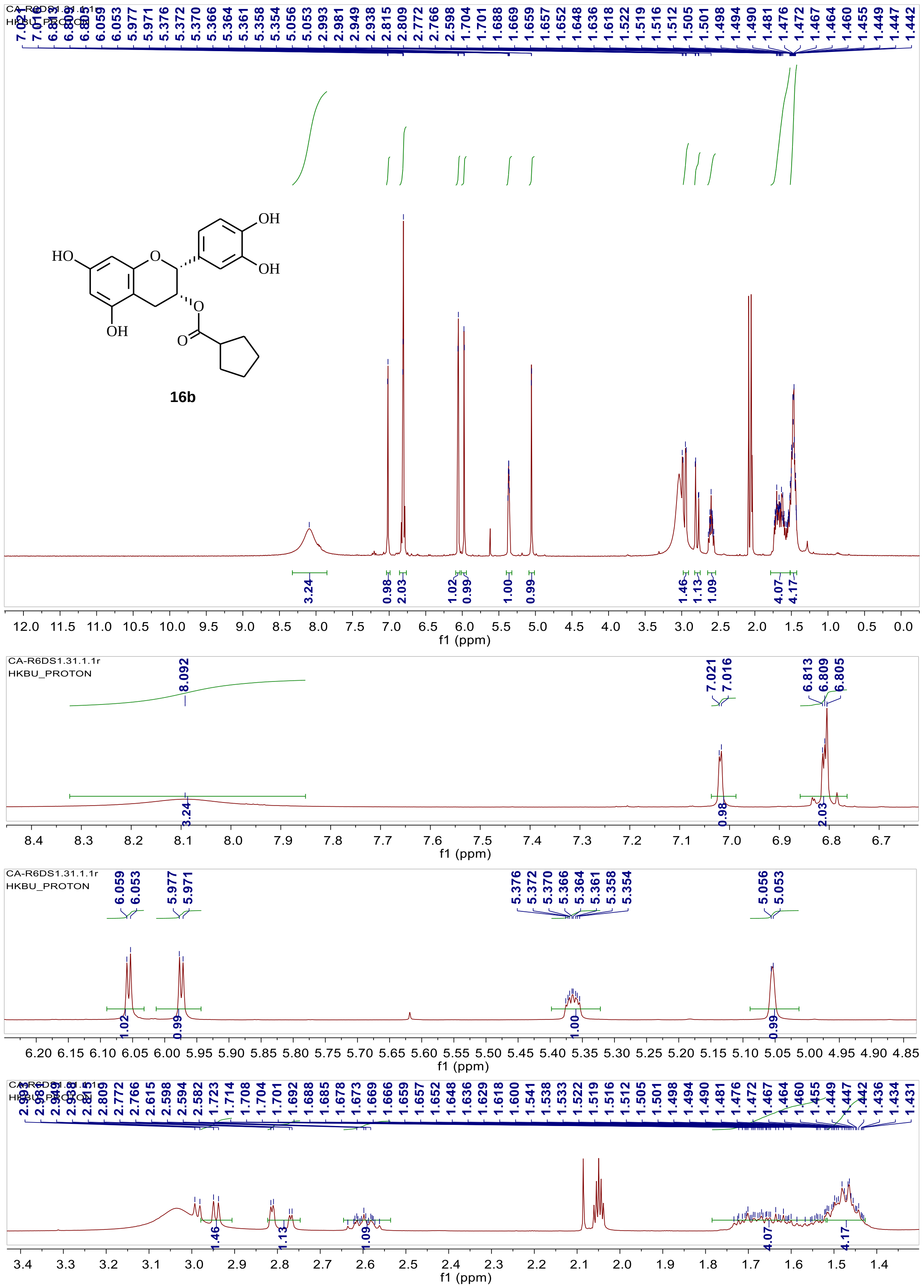

Figure S33. ^{1}H NMR spectrum of **16b** in acetone-d$_6$ (400 MHz).

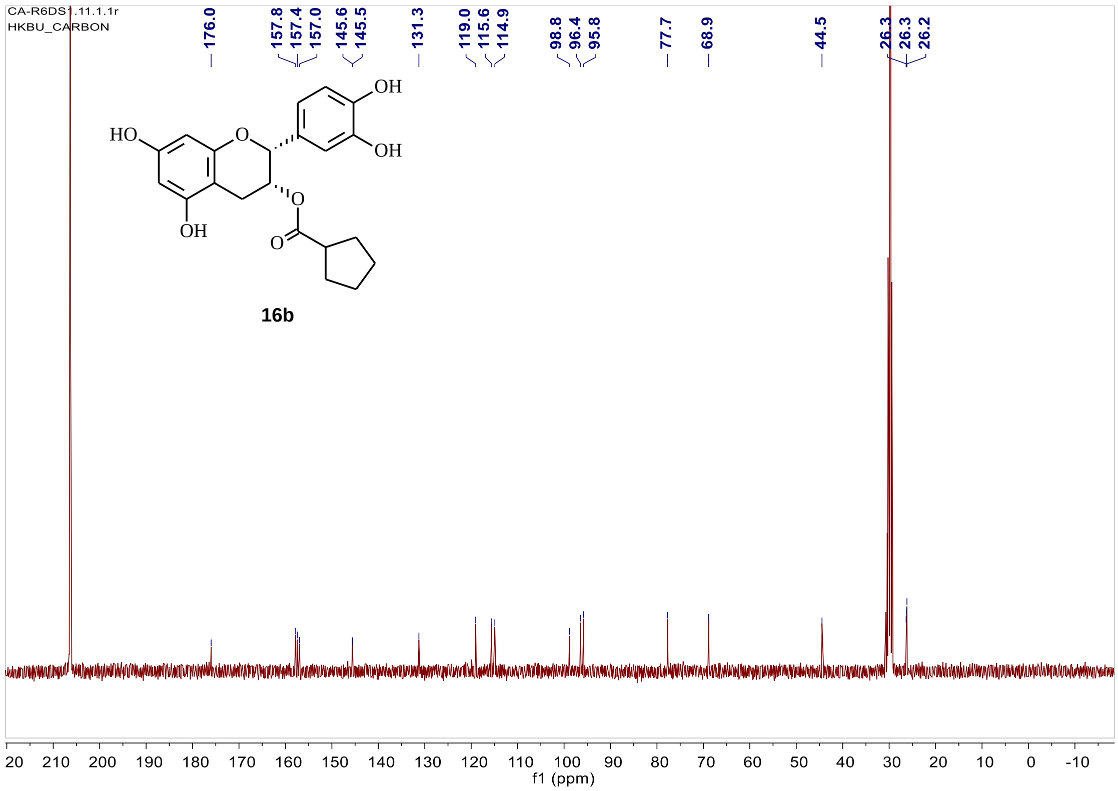

Figure S34. ^{13}C NMR spectrum of **16b** in acetone-d_6 (100 MHz).

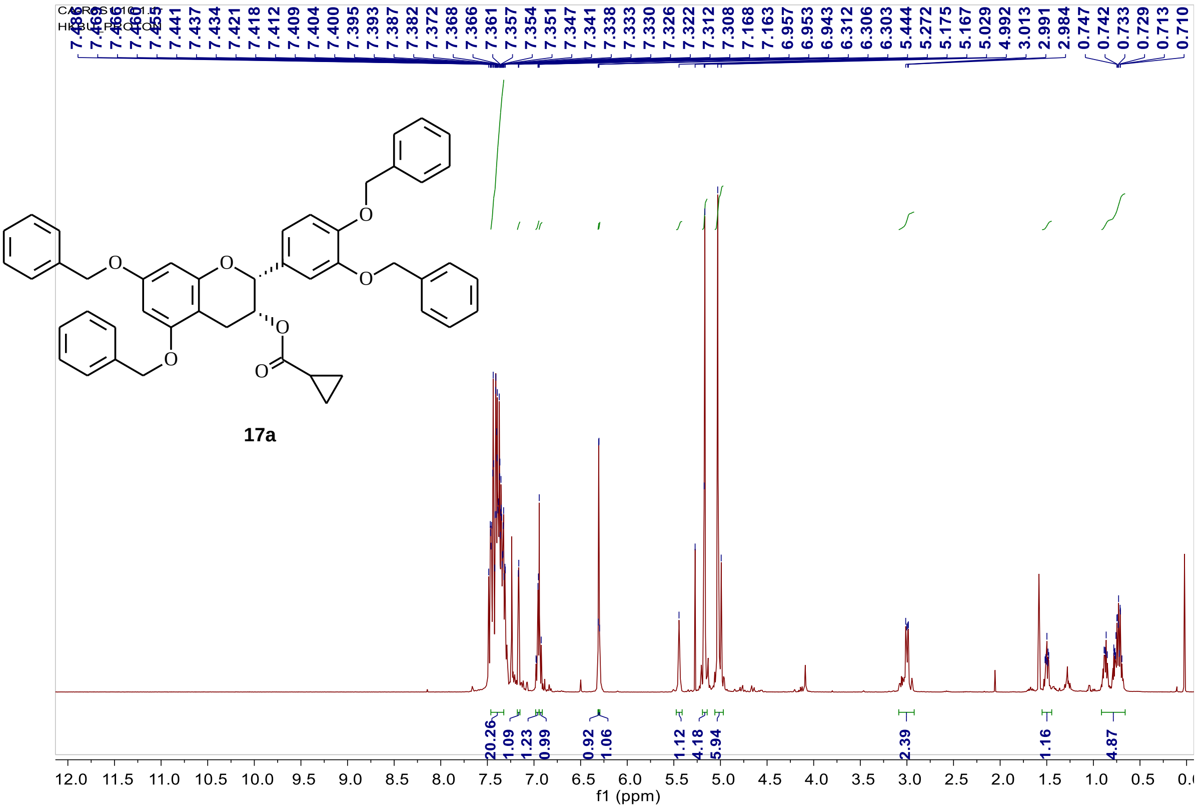

Figure S35. ^{1}H NMR spectrum of **17a** in $CDCl_3$ (400 MHz).

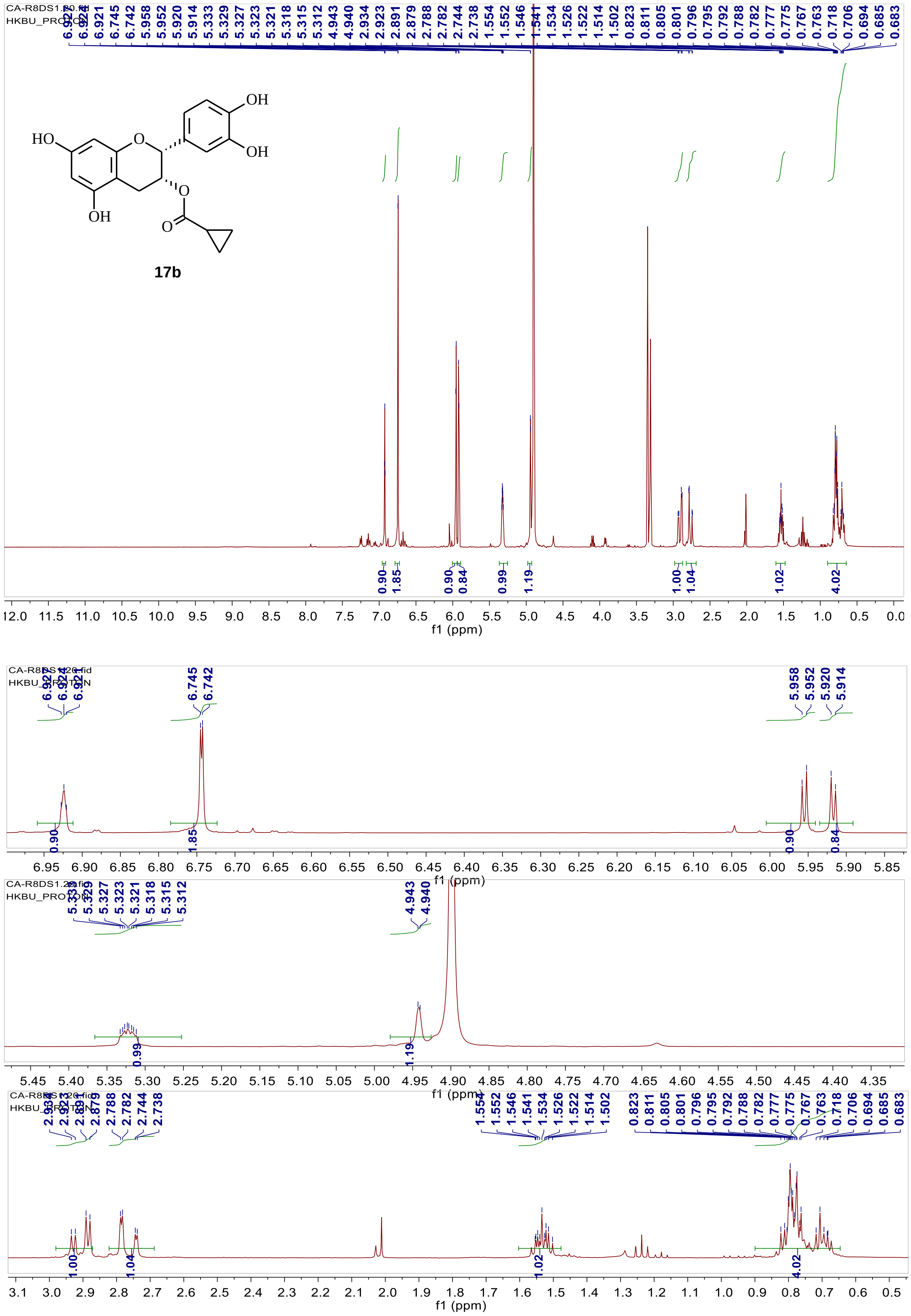

Figure S36. ^{1}H NMR spectrum of **17b** in $CDCl_3$ (400 MHz).

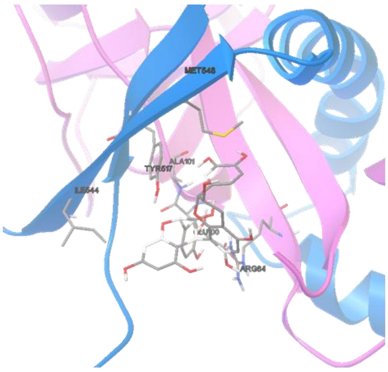

Procyanidin B2 (**1**)

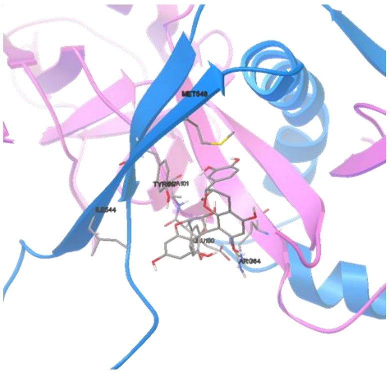

Procyanidin B1 (**6**)

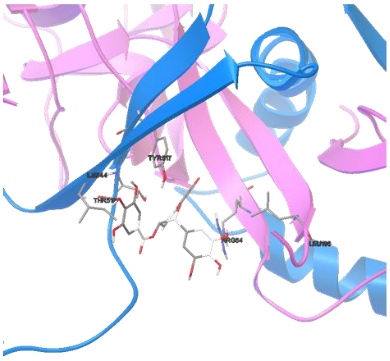

EGCG (8)

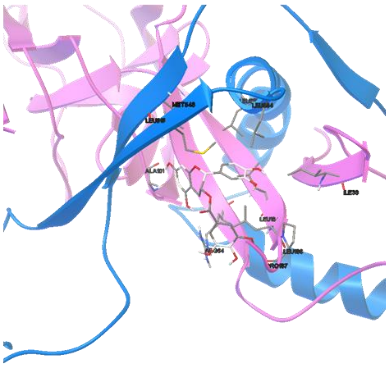

ECG (9)

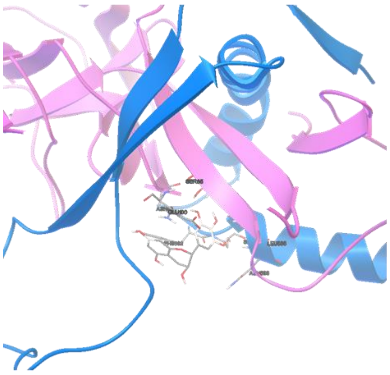

Gallocatechin (10)

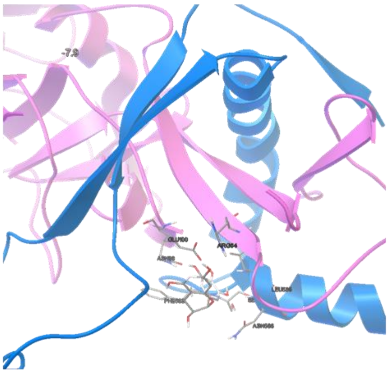

Epigallocatechin (11)

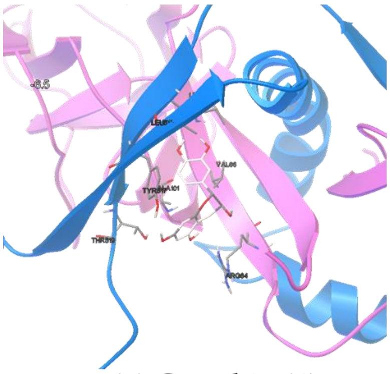

(+) Catechin (4)

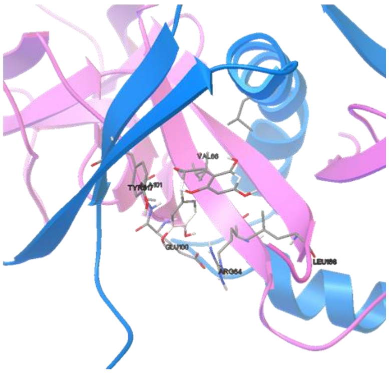

(-) Epicatechin (5)

Figure S37. Binding modes of compounds docked to Ebola virus glycoprotein crystal structure 5JQ7.

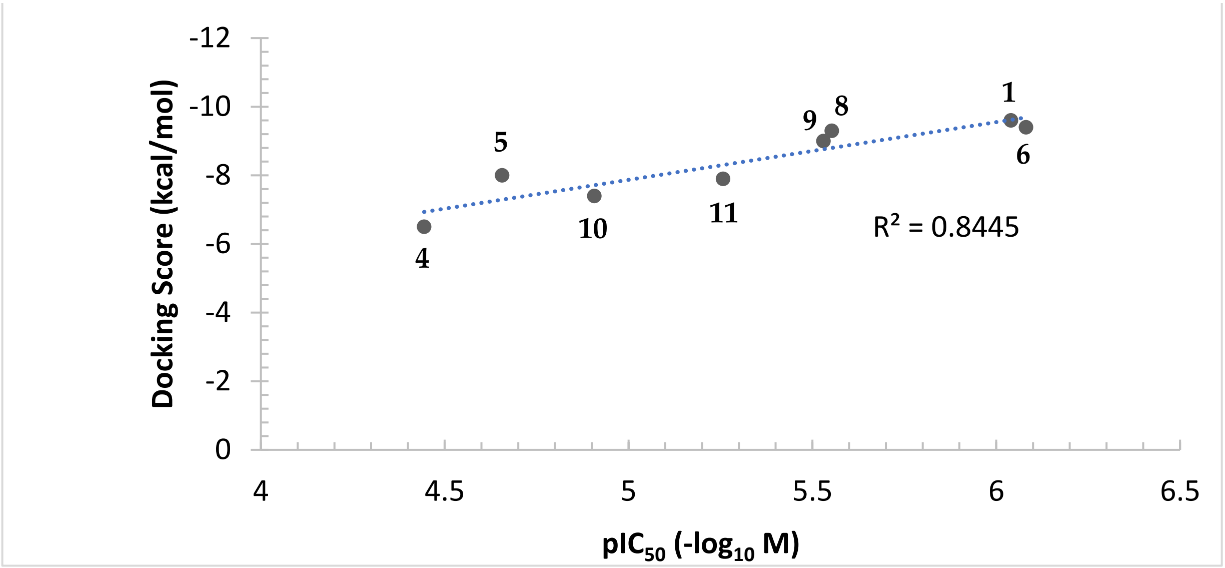

Figure S38. The correlation between the docking score and the IC_{50} values for B-type procyanidins, flavan-3-ols and their analogues. The numbers on the dotted line corresponds to their respective compound numbers (Pearson correlation coefficient r = -0.919, $p < 0.01$).

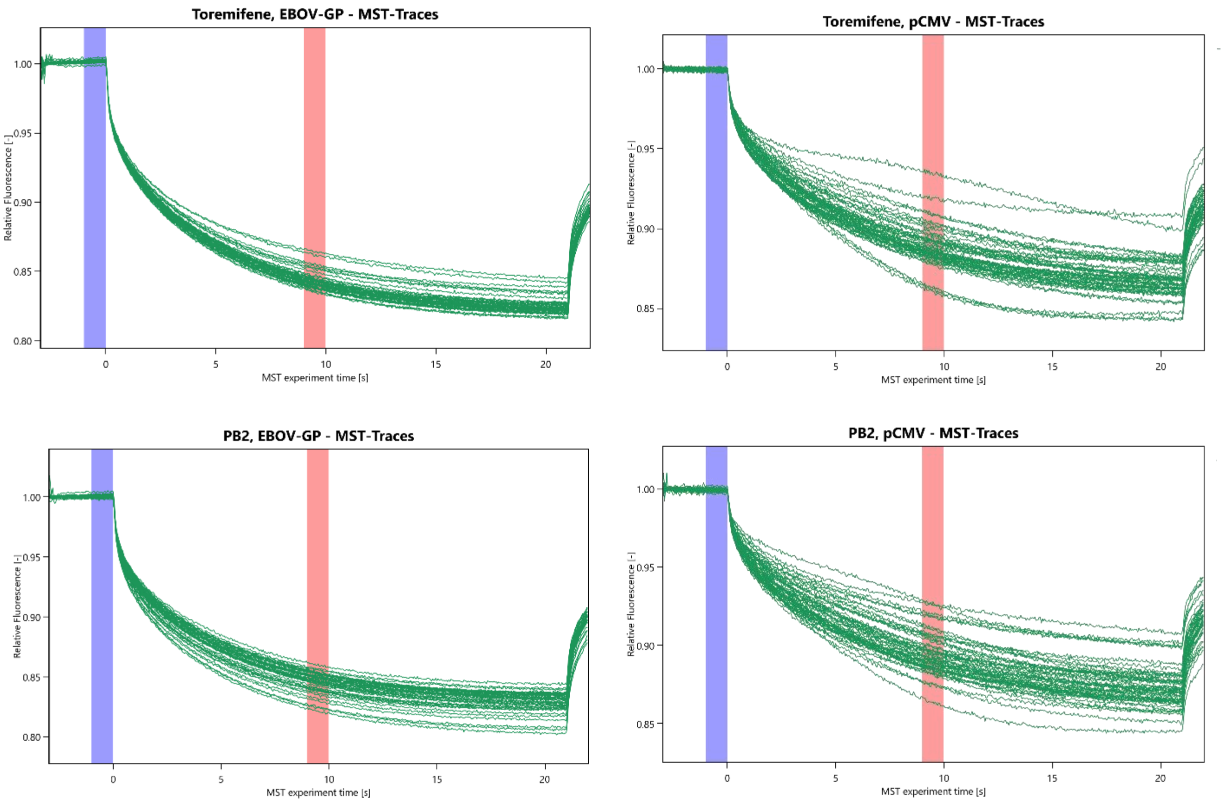

Figure S39. Thermographs of toremifene or procyanidin B2 (**1**) (labeled as PB2) in PBST/M-PER buffer bound to the target protein EBOV-GP. The cold region is set to 0 s (blue) and the hot region is to 10 s (red) for determination of the K_d of the interaction.

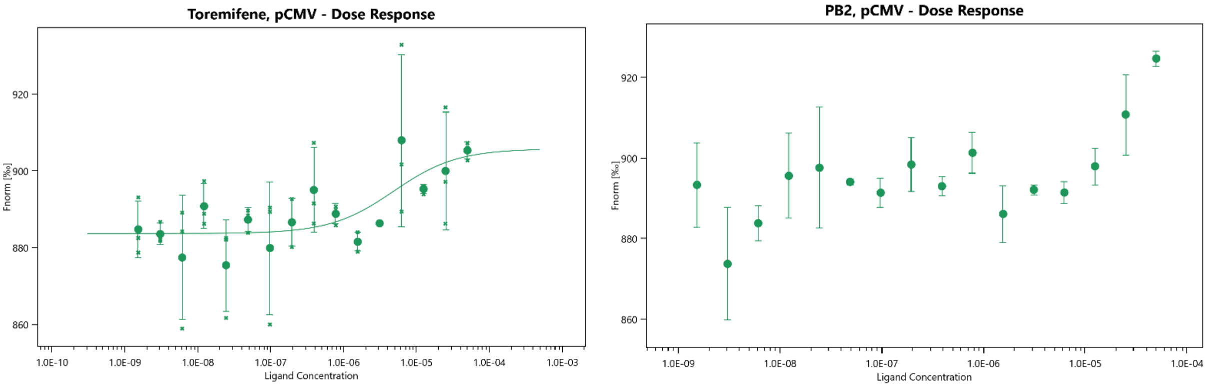

Figure S40. Dose-response curve for the binding interaction between pCMV (negative control) and toremifene/procyanidin B2 (**1**) (PB2).

Table S6. Dataset overview of MST experiments for toremifene and procyanidin B2 (**1**).

**Target Name**	EBOV-GP C-His	pCMV C-His	EBOV-GP C-His	pCMV C-His
**Target Concentration (nM)**	25	25	25	25
**Ligand Name**	Toremifene	Toremifene	Procyanidin B2	Procyanidin B2
**Ligand Concentration (nM)**	$1.53 – 5\times10^4$	$1.53 – 5\times10^4$	$1.53 – 5\times10^4$	$1.53 – 5\times10^4$
**Number of experiments**	3	3	3	3
**Excitation Power**	30%	30%	30%	30%
**MST Power**	40%	40%	40%	40%
**Temperature**	25.0°C	25.0°C	25.0°C	25.0°C
**K_d (M)**	2.1406×10^{-5}	–	1.299×10^{-5}	–
**K_d Confidence (M)**	6.4005×10^{-6} – 7.1589×10^{-5}	–	5.5968×10^{-6} – 3.0148×10^{-5}	–
**Signal to Noise**	12.288503	–	13.518011	–
